# Brain-Inspired Multi-Pathway Motion Decision-Making for Obstacle Avoidance of Humanoid Arms

**DOI:** 10.3390/biomimetics11070469

**Published:** 2026-07-05

**Authors:** Zhengyu Liu, Jiahao Chen

**Affiliations:** 1 The State Key Laboratory of Multimodal Artificial Intelligence Systems, Institute of Automation, Chinese Academy of Sciences, Beijing 100190, China; 2 The School of Artificial Intelligence, University of Chinese Academy of Sciences, Beijing 100049, China

**Keywords:** multi-pathway, obstacle avoidance, decision-making, motion planning, humanoid arm

## Abstract

Achieving rapid and accurate obstacle avoidance in complex and dynamic environments remains a significant challenge for robots. To enhance the adaptability and flexibility of humanoid arms for obstacle avoidance, a brain-inspired multi-pathway motion decision-making method is proposed to modulate rational planning and habitual actions of humanoid arms. Firstly, a novel framework integrating both a slow and a fast pathway is designed for motion decision-making tasks. Imitating the rational planning function of the prefrontal cortex, the slow pathway employs an improved planning approach based on Real-Time Rapidly exploring Random Tree Star (RT-RRT^*^) to execute deliberate decisions, along with an improvement in planning via the Smart technique and the high-efficiency neighbor searching method. Meanwhile, mimicking the habitual responses governed by the striatum, the fast pathway utilizes an action model trained by Soft Actor-Critic to make quick and habitual motions. The model in the fast pathway is also used to guide the sampling strategy in the slow pathway. Moreover, to facilitate the integration and smooth transition between the two pathways, an emotional neural network is designed as the modulation module with inspiration from the structure and function of the amygdala. Based on body and obstacle information, the network generates emotional signals to modulate the involvement degree of the two pathways before each decision-making process. Experimental results demonstrate that the proposed multi-pathway framework achieves a higher obstacle-avoidance success rate than existing methods while generating motion characteristics that are consistent with certain aspects of human obstacle-avoidance behavior.

## 1. Introduction

Robots are increasingly deployed in complex and dynamic environments, ranging from industrial automation to human–robot collaboration (HRI) scenarios [1]. In such settings, robots are required not only to execute predefined tasks but also to adapt their motion safely and efficiently in the presence of static and dynamic obstacles. Therefore, this field requires the support of robot motion decision-making algorithms [2], which demand that robots perform effective motion planning, dynamically adjust actions in response to environmental changes, and handle unpredictable obstacle intrusions within the workspace [3]. However, achieving robust collision avoidance and flexible motion adaptation in complex unstructured environments remains a significant challenge.

Over the past decades, numerous methods have been proposed to address robot motion decision-making problems. Among them, hierarchical planning has emerged as a practical and effective framework, which decomposes motion generation into high-level global planning and low-level local trajectory adjustment, thereby providing a structured solution for complex tasks [3]. Such a strategy has demonstrated promising performance in a variety of robotic applications, particularly for systems with relatively low-dimensional action spaces, such as mobile robots [4]. However, for robotic systems with high-dimensional configuration spaces, such as humanoid arms, maintaining both planning efficiency and real-time adaptability remains challenging [5]. These challenges become more pronounced in highly dynamic environments, where rapid replanning and emergency reactions are often required.

Specifically, global motion planning methods based on sampling [6], convex optimization [7], and numerical optimization [8] have been widely studied for high-dimensional robotic systems. Numerous efforts have been devoted to improving their performance in dynamic environments through techniques such as dynamic replanning [6], chance-constrained planning [9], and search tree pruning [10]. Despite these advances, such methods often rely on iterative search or optimization procedures and may exhibit limited responsiveness when confronted with sudden environmental changes or unexpected obstacle intrusions, particularly under strict real-time requirements.

Within hierarchical frameworks, global motion planning is commonly achieved through sampling-based, convex optimization-based, or numerical optimization-based methods [6,7,8]. These approaches are capable of generating collision-free motions while considering kinematic and dynamic constraints, making them suitable for high-dimensional robotic systems. To improve adaptability in dynamic environments, numerous extensions have been proposed, including dynamic replanning [6], chance-constrained planning [9], and search-tree pruning [10]. Despite these advances, global planning methods generally rely on iterative search or optimization procedures, which may limit their responsiveness when confronted with sudden environmental changes or unexpected obstacle intrusions under strict real-time requirements.

At the local level, motion adaptation and obstacle avoidance have traditionally been addressed through trajectory generation and optimization techniques, ranging from interpolation-based methods to time-optimal trajectory optimization [11,12]. While these methods can produce smooth and feasible motions, their computational cost often increases significantly with problem complexity, limiting their real-time responsiveness [8]. In recent years, reinforcement learning (RL) has emerged as a promising alternative for reactive obstacle avoidance in dynamic environments [13]. Related studies have explored various strategies to improve obstacle avoidance performance, including adaptive observation mechanisms and tailored reward designs [14,15]. By learning directly from interaction experience without requiring explicit environmental models, RL-based methods can provide rapid responses to environmental changes. However, complex motion planning tasks remain challenging for RL alone due to sparse reward signals, training instability, and limited generalization ability. Consequently, there remains a need for motion decision-making frameworks that can simultaneously achieve efficient motion planning, real-time trajectory adaptation, and reliable emergency obstacle avoidance.

These studies suggest that neither planning-based nor learning-based methods alone may be sufficient to achieve robust motion decision-making in complex environments. Therefore, an effective motion decision-making framework should combine the deliberative capability of planning with the adaptability of learned behaviors, enabling both efficient long-term motion generation and reliable emergency obstacle avoidance.

Meanwhile, biological motor decision-making systems in humans provide additional inspiration for designing adaptive robotic control architectures. Human behavior exhibits a dual-process organization, where deliberate, goal-directed planning and fast, habitual reactions are coordinated to ensure both flexibility and efficiency in dynamic environments. Previous studies have demonstrated that leveraging insights from human brain mechanisms is a viable approach to enhancing the human-like capabilities and task performance of robots [16,17,18]. Relevant research has achieved improvements in robot motion capabilities by mimicking the structures and functions of the motor cortex [19], cerebellum [20], and amygdala [21]. However, there is relatively limited brain-inspired work addressing robot motion decision-making in obstacle environments.

In this article, inspired by the multi-pathway mechanism of the human brain, a novel multi-pathway robot motion decision-making framework is proposed to incorporate both fast and slow pathways. Drawing insights from the roles of the prefrontal cortex (PFC), striatum, and amygdala in multi-pathway mechanisms, the framework combines the promising planning and action method at present in a biologically plausible way, enhancing the adaptability and flexibility of robots in dynamic environments with obstacles. Specifically, the main contributions of this article are listed as follows.

1. A novel brain-inspired multi-pathway motion decision-making framework is proposed for humanoid arms operating in complex environments with static and dynamic obstacles. The framework integrates sampling-based planning as a deliberative slow pathway and model-free reinforcement learning (MFRL) as a reactive fast pathway. Furthermore, the learned obstacle-avoidance behavior of the fast pathway is incorporated into the sampling process to guide global planning in high-dimensional spaces.
2. An improved hierarchical planning scheme from path generation to trajectory execution is developed to enhance real-time planning performance. Specifically, the sampling-based planner is accelerated through Smart-based path refinement and an efficient nearest-neighbor search strategy, improving planning efficiency while maintaining solution quality.
3. An emotion-inspired modulation mechanism is introduced to regulate the interaction between the slow and fast pathways. By generating emotion-like signals in response to environmental stimuli, the proposed mechanism adaptively adjusts the influence of fast-pathway guidance, facilitating a smooth integration and transition between deliberative planning and reactive obstacle avoidance.

The rest of this article is organized as follows. Section 3 introduces the necessary preliminaries of the framework, including the problem definition and brief descriptions of the promising real-time sampling-based planning and MFRL methods. Section 4 illustrates the proposed framework in detail. Section 5 presents the evaluation results of our methods in simulations. Finally, Section 6 concludes this article. For the readers’ convenience, nomenclature tables are provided in Appendix A.

## 2. Related Works

### 2.1. Hierarchical Planning and Reinforcement Learning for Robot Motion Decision-Making

In robot motion decision-making, hierarchical planning has been widely adopted as a mainstream framework. A typical approach is to combine a global planner with a local trajectory optimizer based on numerical optimization or interpolation [22]. Although global planners provide asymptotically optimal and collision-free paths, the combination of global planning and optimization-based trajectory generation incurs substantial computational costs, particularly in high-dimensional configuration spaces. Traditional numerical optimization methods for redundant manipulators often suffer from the curse of dimensionality, resulting in limited scalability and reduced computational efficiency [23]. Moreover, coupling sampling exploration with local trajectory optimization may lead to solver failures in complex environments, requiring repeated optimization and increasing planning latency [24]. In dynamic and unstructured environments, local reactive controllers within conventional hierarchical frameworks often lack adaptive parameter adjustment and predictive capabilities for dynamic obstacles, relying primarily on immediate reactive avoidance behaviors [25]. As a result, the computational burden and limited adaptability of traditional local optimization methods make it difficult to satisfy the real-time requirements of safe navigation in highly dynamic environments.

To address the latency associated with traditional local optimizers, MFRL has been increasingly introduced into hierarchical architectures due to its rapid inference and robustness against dynamic disturbances. MFRL has been widely applied in robot path planning [15], demonstrating effectiveness in enabling rapid reactive responses and generating safe alternative trajectories [26]. Currently, the integration of classical planning and RL predominantly follows two paradigms. The first employs a planner–tracker structure, where a global planner generates a sequence of waypoints and a trained RL policy serves as a low-level controller to track the planned path while performing local obstacle avoidance [27]. The second utilizes RL to learn heuristic distributions or value functions that guide and accelerate the sampling process of the global motion planner [28]. Overall, through extensive offline learning, MFRL can implicitly process high-dimensional kinodynamic constraints, which helps conventional methods with emergencies [29].

Despite the performance improvements achieved by these hybrid architectures, most existing frameworks still rely on fixed hierarchical structures or predefined switching strategies. When faced with sudden and highly urgent situations, such rigid coordination mechanisms often struggle to dynamically balance long-horizon planning and short-term reactive behaviors. Consequently, they may exhibit oscillatory switching behavior or generate suboptimal decisions during emergency obstacle avoidance. Addressing this limitation requires a context-aware coordination mechanism that can assess environmental urgency and dynamically regulate the interaction between sampling-based planning and reinforcement learning. This challenge motivates the development of the brain-inspired multi-pathway architecture and emotion-inspired regulation mechanism proposed in this work.

### 2.2. Human Multi-Pathway Decision-Making Mechanism

The high adaptability and great flexibility of human motion decision-making in complex environments have attracted great attention, as they are associated with the multi-pathway mechanism in the human brain, as suggested in relevant neuroscience research. Specifically, regions including PFC, the striatum, and the amygdala in the human brain function as an integrated circuit orchestrating motor decision-making processes, divided into slow and fast pathways. The PFC is considered the center of planning [30], playing a central role in deliberate, rational, and analytical decision-making processes, primarily within the slow pathway. In contrast, the striatum is critical for the control of habitual action [31], which dominates the process of the acquisition and application of motor skills from experience and intuition. In addition, the striatum is also crucial for planning-guided action [32], where the dorsal striatum contributes to plan selection and initiation of the cortex through the integration of motivational/emotional information [33]. It is worth noting that these two pathways are modulated by emotional signals related to stress and fear from the amygdala [34].

**Figure 1:**
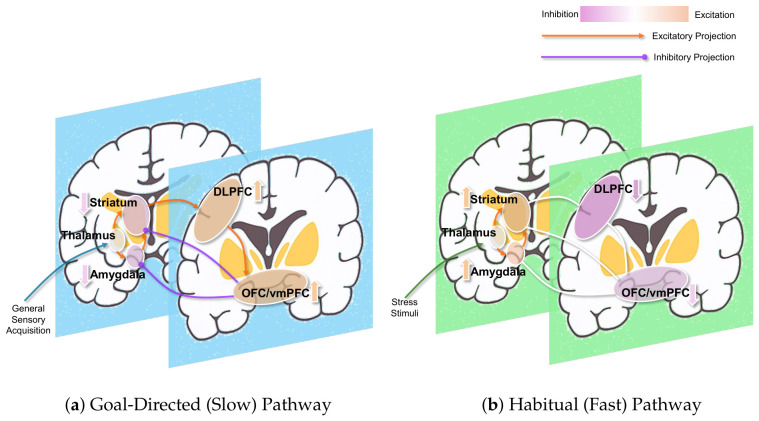
Neural mechanisms of multi-pathway decision-making in the human brain. (**a**) Under normal conditions, the goal-directed slow pathway dominates. The dlPFC exerts top-down suppression on the striatum-mediated habitual responses, while the OFC/vmPFC maintains emotional stability by regulating the amygdala. (**b**) Under stressful conditions induced by external threats, the habitual fast pathway is activated. Heightened amygdala activity inhibits the dlPFC and impairs the regulatory capacity of the OFC/vmPFC, leading to an increase in rapid, habitual responses. Upward arrows indicate neural activation or functional up-regulation, whereas downward arrows denote functional inhibition or down-regulation. Abbreviations: dlPFC, dorsolateral prefrontal cortex; OFC, orbitofrontal cortex; vmPFC, ventromedial prefrontal cortex.

As shown in Figure 1, in slow-pathway decision-making, the dorsolateral prefrontal cortex (dlPFC) plays a pivotal role in goal-directed behavior by exerting top-down control that suppresses habitual responses mediated by the striatum, which facilitates flexible, model-based decision-making processes [35]. Meanwhile, the ventromedial prefrontal cortex (vmPFC) modulates amygdala activity through functional connectivity, thereby maintaining emotional stability and preventing excessive reactivity [36]. In contrast, under fast-pathway decision-making conditions, external threats inducing survival stress lead to the activation of the amygdala, which subsequently inhibits the planning function of the dlPFC, leading to a reduction in goal-directed behavior and an increase in habitual responses [37]. Moreover, stress adversely affects the orbitofrontal cortex (OFC) and vmPFC, further compromising their regulatory capacities. This degradation enhances amygdala activity, resulting in heightened emotional responses and reduced behavioral flexibility. In summary, the brain’s multi-pathway mechanism enables humans to excellently adapt to dynamic environments.

## 3. Preliminaries

### 3.1. Problem Definition

The problem of robot motion decision-making in complex environments can be generalized into the tasks of planning and motion in consideration of obstacles. Let us denote the planning state space by $𝒳\subseteq R^{m},m\in N$. Generally, 𝒳 is bounded due to physical constraints. ${𝒳}_{\mathrm{obs}}\subseteq 𝒳$ denotes the set of all states in 𝒳 that are interfered by obstacles, leaving ${𝒳}_{\mathrm{free}}=𝒳\setminus {𝒳}_{\mathrm{obs}}$ as the free space. Both ${𝒳}_{\mathrm{free}}$ and ${𝒳}_{\mathrm{obs}}$ are changing constantly in environments with dynamic obstacles. States in the environment are denoted by x∈𝒳. For example, the initial state of the agent is represented by the state $x_{\mathrm{init}}$, and the goal by $x_{\mathrm{goal}}$, which are both provided to the planner as inputs. Similarly, the state of the agent is represented by $x_{a}$, serving as the starting point for planning. At start time $t_{0}$, $x_{a}=x_{\mathrm{init}}$.

Generally, the goal of planning tasks is to obtain a trajectory that enables the robot to move to the target. Let $\sigma \left(s\right):\left[0,1\right]\to 𝒳$ represent a trajectory, and Σ denote the set of all trajectories. The obstacle avoidance condition requires $\forall s\in \left[0,1\right],\;\;\sigma \left(s\right)\in {𝒳}_{\mathrm{free}}$.

To evaluate the trajectories, a cost function is usually designed to bring relevant information about optimization objectives and constraints into consideration. Given the cost function c(σ):Σ→R, the optimal trajectory planning problem can be defined as finding an obstacle avoidance trajectory $\sigma^{*}$, which connects the initial state and the target state and minimizes the cost function value, as shown in (1):

(1) $$\begin{array}{c} \sigma^{*}=\arg\;\min_{\sigma \in \Sigma }\;\;c\left(\sigma \right)\;\;\;\;\;\;\\ s.t.\;\left\{\begin{array}{c} \sigma \left(0\right)=x_{\mathrm{init}},\\ \sigma \left(1\right)=x_{\mathrm{goal}},\\ \sigma \left(s\right)\in {𝒳}_{\mathrm{free}},\;\;s\in \left[0,1\right]. \end{array}\right. \end{array}$$

When the joint space of the humanoid arm is defined as the planning state space, the problem then implicates a sequence of actions to find which minimizes the cost of the agent to reach the goal while avoiding collisions.

Given that the situation of obstacles is changing over time, the trajectory needs to be solved online in real time and adjusted based on the current solution to ensure feasibility.

### 3.2. Sampling-Based Method

In practice, solving the entire trajectory directly faces a significant computational burden, which can hardly meet real-time requirements. Hierarchical approaches can be involved to accelerate the process, whose core idea is to plan the path to the target first, and then obtain a trajectory through the path points via other methods.

Let the finite sequence $\sigma_{p}:\langle x_{0},x_{1},\ldots ,x_{k}\rangle$ denote a path from $x_{0}$ to $x_{k}$, $\sigma_{p}\subset 𝒳$. $d(x_{i},x_{j}):𝒳\times 𝒳\to R$ is a metric function on 𝒳, representing the distance from $x_{i}$ to $x_{j}$. When 𝒳 is in Cartesian coordinate space, usually $d\left(\cdot ,\cdot \right)$ can be a Euclid distance (or another type). Then, the cost function of the path is

(2) $$c\left(\sigma_{p}\right)=\sum_{i=0}^{N-1}d\left(x_{i},x_{i+1}\right).$$

The target of path planning is to find and maintain a collision-free path $\sigma_{p}\subset {𝒳}_{\mathrm{free}}$ between $x_{a}$ and $x_{\mathrm{goal}}$ to minimize the cost function, which can be achieved via sampling-based methods. The class of Rapidly exploring Random Tree (RRT) algorithms is known as a kind of efficient sampling-based method providing quick solutions for complex and high-dimensional planning space, which is suitable for real-time tasks.

RRT-class algorithms achieve path planning through the construction of search trees. Specifically, let $ㄒ=\left(𝒱,E\right)$ be a search tree, where 𝒱⊂𝒳 denotes the set of nodes and E:𝒱×𝒱→R represents the weighted set of edges. The weight represents the cost of the edge between two nodes. $x_{\mathrm{root}}$ is the root node of ㄒ. Since ㄒ is a tree, there exists a unique path between any two nodes in ㄒ. Therefore, we denote the path from $x_{\mathrm{root}}$ to node $x_{i}$ as $\sigma_{p}\left(x_{i}\right)$, and its cost is given by $c\left(x_{i}\right)=c\left(\sigma_{p}\left(x_{i}\right)\right)$.

By extending ㄒ in the search space, RRT-class algorithms can rapidly find an initial path towards the goal, with a notable instance being the Rapidly exploring Random Tree Star (RRT^∗^) algorithm [38]. RRT^∗^ optimizes the selection of a parent for new nodes and introduces a Rewire step, thereby achieving probabilistic completeness of path planning and asymptotic optimality of solution cost. Based upon this foundation, the Real-Time Rapidly exploring Random Tree Star (RT-RRT^*^) [39] is proposed, which enables real-time path solving and adjustment. The key steps of the growth process of ㄒ are described as follows.

1. Sample: The function $x_{\mathrm{rand}}\leftarrow \text{Sample}\left(f\left(x_{a},ㄒ\right)\right)$ generates a random state $x_{\mathrm{rand}}$ according to a specific sampling distribution *f*, such as a uniform distribution on 𝒳 in a simple situation.
2. Nearest: The function $x_{\mathrm{nearest}}\leftarrow \text{Nearest}\left(ㄒ,x_{\mathrm{rand}}\right)$ returns the nearest node from ㄒ to $x_{\mathrm{rand}}$ according to the metric function $d\left(\cdot ,\cdot \right)$, i.e.,
  (3) $$\text{Nearest}\left(ㄒ,x_{\mathrm{rand}}\right)≐\arg\;\min_{x\in 𝒱}\;d\left(x,x_{\mathrm{rand}}\right).$$
3. Steer: The function $x_{\mathrm{new}}\leftarrow \text{Steer}\left(x_{\mathrm{nearest}},x_{\mathrm{rand}}\right)$ generates a new node $x_{\mathrm{new}}$ at a step size $\rho_{s}$ from $x_{\mathrm{nearest}}$ along the direction from $x_{\mathrm{nearest}}$ to $x_{\mathrm{rand}}$ ($x_{\mathrm{rand}}$ itself if $d\left(x_{\mathrm{nearest}},x_{\mathrm{rand}}\right)<\rho_{s}$), which is prepared to be added to the tree ㄒ according to certain rules. $x_{\mathrm{new}}$ can be selected on the line of the two input nodes in simple situations.
4. Near, ChooseParent, and InsertNode: Instead of directly connecting $x_{\mathrm{nearest}}$ and $x_{\mathrm{new}}$, RRT^∗^ re-selects the parent $x_{\min}$ for $x_{\mathrm{new}}$ in its neighbor ${𝒳}_{\mathrm{near}}$ within a distance $\rho_{N}$ from $x_{\mathrm{new}}$, i.e.,
  (4) $$\text{Near}\left(ㄒ,x_{\mathrm{new}}\right)≐\left\{x\in 𝒱|d\left(x,x_{\mathrm{new}}\right)<\rho_{N}\right\},$$
  which further minimizes $c(x_{\mathrm{new}})$. Then, $x_{\mathrm{new}}$ can be inserted into ㄒ, connecting to $x_{\min}$ as its parent.
5. Rewire: Rewire is applied to the areas where the tree ㄒ undergoes changes, including the neighbors of $x_{\mathrm{new}}$, the changing $x_{\mathrm{root}}$, and nodes near dynamic obstacles, aiming to renew their parents when a lower cost can be achieved by passing through another new node instead of their old parent. In RT-RRT^*^, Rewire includes two steps: Rewire_new_ and Rewire_root_. The former checks the nodes near $x_{\mathrm{new}}$ and dynamic obstacles, ensuring the feasibility and asymptotic optimality of path planning. The latter rewires the areas near $x_{\mathrm{root}}$ when it changes, maintaining the consistency of path planning while making minor modifications to the tree structure. Algorithm 1 describes the process of the Rewire_new_ step for an instance where Rewire_root_ is similar to it while updating from $x_{\mathrm{root}}$ using $Q_{s}$. The use of queues $Q_{r}$ and $Q_{s}$ enables the algorithm to update the tree ㄒ in a breadth-first search (BFS) manner, ensuring that when the decision time in each round is insufficient for complete updates, nodes close to the center can be prioritized for updating.

**Algorithm 1** Rewire_new_: Rewire from new node
  1:**Inputs:** $T,Q_{r},x_{\mathrm{new}}$  2:

$Q_{r}.\text{InsertToFirst}\left(x_{\mathrm{new}}\right)$

  3:**while** $Q_{r}\neq \emptyset$ **and** $t_{\mathrm{plan}}>0$ **do**  4:      $x_{r}\leftarrow Q_{r}.\text{Dequeue}\left(\right)$, ${𝒳}_{\mathrm{near}}\leftarrow \text{Near}\left(T,x_{r}\right)$  5:      **for** $x_{\mathrm{near}}\in {𝒳}_{\mathrm{near}}$ **do**  6:          **if** $c\left(x_{r}\right)+d\left(x_{r},x_{\mathrm{near}}\right)<c\left(x_{\mathrm{near}}\right)$ **then**  7:             $x_{\mathrm{near}}.\mathrm{Parent}\leftarrow x_{r}$, $Q_{r}.\text{Enqueue}\left(x_{\mathrm{near}}\right)$  8:          **end if**  9:      **end for**10:
**end while**
11:**return** $ㄒ,Q_{r}$


To achieve online planning, RT-RRT^*^ constrains the period of each update cycle $T_{d}$, enabling it to promptly incur path planning after each decision time step. Meanwhile, as the agent moves, $x_{\mathrm{root}}$ changes along the planned path to keep the agent near the tree root and moving toward it. At the end of each duration, RT-RRT^*^ provides a planned path $\sigma_{p}=\langle x_{\mathrm{root}},x_{1},\ldots ,x_{k}\rangle$ to the goal (if reached) or the nearest position in ㄒ to the goal through the PathPlan step, combined with other trajectory planning methods to generate required action.

### 3.3. Model-Free Reinforcement Learning Method

The parameterized models trained by reinforcement learning (RL) show notable computational efficiency during online planning, such as the neural network (NN), which is suitable for robotic real-time dynamic obstacle avoidance. To introduce RL, it is essential to first expand the robot motion decision-making model as a Markov Decision Process (MDP) defined by a tuple M=(𝒮,𝒜,P,r,γ), in which $𝒮=\left[𝒳,Ƶ\right]$ is the state space, which usually contains more information than 𝒳, such as states of the environment and obstacles. 𝒜 is the action space. P:𝒮×𝒜×𝒮→[0,1] represents the state transition probability, determining the distribution of the next state $s_{t+1}$ given the current state $s_{t}\in 𝒮$ and action $a_{t}\in 𝒜$. r:𝒮×𝒜→R is the reward function. $\gamma \in \left[0,1\right)$ is the discount factor.

RL aims to derive an optimal policy $\pi^{*}$ by maximizing the cumulative reward. To solve problem (1) in RL, the trajectory σ(s) can be discretized into a sequence $\sigma_{n}$: $\sigma_{n}=\sigma \left(nT_{c}/\left(t_{f}-t_{0}\right)\right)$, where $T_{c}$ denotes the control period, and $t_{0}$ and $t_{f}$ represent the starting and finishing time of the σ execution process. Additionally, the reward function $r(s_{t},a_{t})$ needs to be constructed according to the cost function c(x). r(·) can be heuristic to achieve expedited high training efficiency and enhance agent performance.

Given the difficulty in predicting obstacles within complex dynamic environments and the necessity for generalization across diverse obstacle scenarios, MFRL methods are suitable for learning obstacle avoidance strategies. In recent works, the Soft Actor-Critic (SAC) [40] algorithm has emerged as a promising MFRL method, which exhibits robust performance in high-dimensional MDPs with continuous action space. In SAC, the problem (1) can be reformulated as follows:

(5) $$\pi^{*}=\arg\;\max_{\pi }\;E_{\pi }\left[\sum_{t=0}^{T}r\left(s_{t},a_{t}\right)+\alpha H\left(\pi \right)\right]$$

where H is the entropy introduced by SAC, which quantifies the uncertainty of the policy. By maximizing the entropy, π is encouraged to facilitate the exploration of various actions, thereby enhancing the algorithm’s exploration capability. Additionally, α, denoted as the temperature coefficient, is employed to modulate the emphasis of H, thus adjusting the exploration of π during the learning process.

Based on the actor-critic framework, SAC employs two types of parameterized functions, the critic $Q_{\theta }\left(s_{t},a_{t}\right)$ and the actor $\pi_{\phi }\left(a_{t}|s_{t}\right)$, to approximate the action-value function *Q* and the policy π, where θ and ϕ are the parameters of the critic and actor model, respectively. The critic receives state–action pairs as input and returns a scalar representing the action value, while the actor accepts states as input and outputs a distribution of actions in 𝒜. When practical actions are required, a single sample is drawn from the obtained distribution. The parameters θ and ϕ are updated via the following objective functions:

(6) $$J_{\pi }\left(\phi \right)=E_{s_{t}∼𝒟,a_{t}∼\pi_{\phi }}\left[\alpha \log\;\;\left(\pi_{\phi }\left(\left.a_{t}\right|s_{t}\right)\right)-Q_{\theta }\left(s_{t},a_{t}\right)\right],$$

where $J_{\pi }\left(\phi \right)$ serves as the actor loss function minimized to achieve policy improvement by driving the policy toward higher estimated action values.

(7) $$J_{Q}\left(\theta \right)=E_{\left(s_{t},a_{t}\right)∼𝒟}\left[\frac{1}{2}{\left(Q_{\theta }\left(s_{t},a_{t}\right)-\left(r\left(s_{t},a_{t}\right)+\gamma E_{s_{t+1}∼P}\left[V_{\theta }\left(s_{t+1}\right)\right]\right)\right)}^{2}\right],$$

where $J_{Q}\left(\theta \right)$ represents the critic loss function, corresponding to the soft Bellman residual, which is minimized during the policy evaluation step to accurately estimate state–action values. Here, the soft state-value function $V_{\theta }$ is implicitly parameterized through the soft action-value function $Q_{\theta }$ as follows:

(8) $$V_{\theta }\left(s_{t+1}\right)=E_{a_{t+1}∼\pi }\left[Q_{\theta }\left(s_{t+1},a_{t+1}\right)-\alpha \log\pi \left(a_{t+1}|s_{t+1}\right)\right].$$

𝒟 is the sample space for training, which is typically implemented as an experience replay pool in MFRL. α can also be adaptively adjusted by optimizing the following loss function [41]:

(9) $$J\left(\alpha \right)=E_{a_{t}∼\pi_{\phi }}\left[-\alpha \log\;\;\left(\pi_{\phi }\left(\left.a_{t}\right|s_{t}\right)\right)-H_{0}\right]$$

where $H_{0}=-\dim\left(𝒜\right)$ depends on the dimension of the action space.

By iterative sampling in the environment and optimizing θ, ϕ, and α in the line search method sequentially, a trained actor model for obstacle avoidance actions can be obtained, which can subsequently be utilized for online solving of discrete obstacle avoidance trajectories. Then, $\left\{\sigma_{n}\right\}$ can be solved online.

## 4. Method

### 4.1. Architecture of Brain-Inspired Multi-Pathway Method

**Figure 2:**
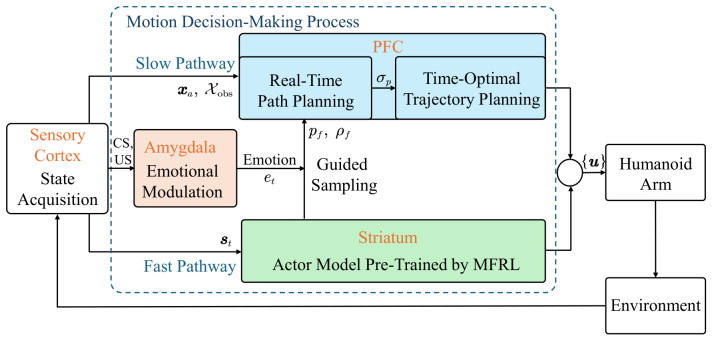
System architecture of the proposed brain-inspired multi-pathway motion decision-making framework. The architecture is functionally inspired by biological decision-making systems. The fast pathway employs a pre-trained model-free reinforcement learning (MFRL) actor policy to provide rapid reactive responses inspired by the striatum, while the slow pathway performs geometric path planning and time-optimal trajectory generation inspired by the planning functions of the prefrontal cortex. A biologically inspired emotional modulation module evaluates environmental collision threats and generates a continuous modulation signal to regulate pathway selection and heuristic guidance from the fast pathway to the slow pathway.

Inspired by the multi-pathway characteristics of human decision-making, this paper proposes a biologically inspired multi-pathway motion decision-making framework for humanoid arms, as illustrated in Figure 2. The framework is motivated by functional principles reported in neuroscience studies, and is developed as an effective architecture for robotic motion decision-making.

Specifically, the framework draws inspiration from three brain regions frequently associated with rapid action selection, deliberative planning, and emotional modulation: the striatum, the prefrontal cortex (PFC), and the amygdala. Correspondingly, the framework consists of three main components: the fast pathway, the slow pathway, and the modulation module. The fast pathway, implemented as an actor model trained via an MFRL method, provides rapid obstacle-avoidance behaviors inspired by habitual action selection processes commonly associated with the striatum. The slow pathway performs real-time sampling-based path planning and trajectory generation, drawing inspiration from the executive planning functions often attributed to the PFC. The modulation module is designed as a biologically inspired emotional regulation mechanism that coordinates the interaction between the two pathways according to the perceived environmental risk.

Upon receiving sensory observations, the modulation module evaluates the environmental state and generates a continuous emotional valence signal that influences pathway selection. Under safe conditions, the slow pathway predominates and performs deliberate long-horizon planning. As the perceived threat from dynamic obstacles increases, the modulation signal gradually increases the influence of the fast pathway on the planning process of the slow pathway. Under highly urgent situations, the fast pathway may temporarily dominate the decision-making process to ensure reactive obstacle avoidance. The selected motion command is then executed by the robot according to the currently active pathway. Based on the selected decision mode, the decision module outputs joint position increment commands to the robot control system, which subsequently drives the actuators to execute the desired motion. The referenced brain regions serve as sources of inspiration for the design of computational modules that capture several qualitative characteristics of biological decision-making, including rapid reaction, deliberative planning, and adaptive pathway modulation.

In the following sections, the design and implementation of the fast pathway, slow pathway, and emotion modulation module are introduced in detail.

### 4.2. The Fast-Pathway Motion Model

The fast pathway aims to emulate the habitual decision-making process of the striatum in the human brain, achieving obstacle avoidance under emergency conditions. Given the evidence of the relationship between the decision-making mechanism of striatum and RL, it is suggested that the function be implemented with the MFRL method. Specifically, the SAC algorithm is chosen as the implementation method for the fast-pathway method due to its advantages such as insensitivity to hyperparameters and relaxed task requirements, which contribute to enhancing the adaptability of robots in obstacle environments. Compared to deterministic alternatives such as Twin Delayed DDPG (TD3), the SAC framework is selected due to its maximum entropy formulation, which offers distinct advantages for the fast pathway. Since SAC optimizes a stochastic policy with entropy regularization, it tends to maintain action diversity and is more likely to cover different evasive behavioral modes in scenarios with unpredictable obstacle trajectories and redundant feasible paths. In contrast, deterministic alternatives optimize a deterministic policy and typically tend to converge toward a single local optimal avoidance strategy, which restricts tactical flexibility. Furthermore, the entropy-regularized objective function of SAC serves as an intrinsic mathematical smoother, helping to mitigate high-frequency joint chattering and generating continuous velocity profiles that align with human-like musculoskeletal compliance. Finally, the probabilistic uncertainty exploration of SAC closely mirrors the adaptive habit-extraction mechanism of the biological striatum, maintaining strong conceptual consistency with our brain-inspired architecture. The goal of training is to teach the agent to avoid dynamic obstacles while reaching the goal. In terms of real-time requirements, the policy is well trained offline before the task.

#### 4.2.1. State Space and Action Space

Generally, apart from robot dynamics, the implementation of collision avoidance capability relies on the agent’s perception of the environment and obstacle information, which may entail robot vision to calculate obstacle distance information necessary for collision avoidance, or ranging methods (like ultrasound or radar) to gather local environmental and obstacle data. In the fast pathway, it is reasonable for us to assume that the robot can dynamically access obstacle positions and the distances between itself and obstacles, which serve as inputs of the algorithm. Therefore, elements in the state space 𝒮 are defined as

(10) $$s≐\left[q,\dot{q},p_{\mathrm{goal}},d_{o},p_{rd},p_{od}\right],$$

consisting of the joint position q, joint angle velocity $\dot{q}$, and goal position $p_{\mathrm{goal}}$, as well as the minimum distance between the robot and the obstacle in task space $d_{o}$, and the two endpoints of it ($p_{rd}$ on the robot and $p_{od}$ on the obstacles). To handle varying numbers of dynamic obstacles without altering the static input layer dimensionality of the SAC neural network, a selective attention mechanism is implemented. At each time step, the state representation vector filters the environment and maps the relative kinematics of exclusively the single critical obstacle that is the nearest hazard to the robot’s links, thereby ensuring a constant input dimension regardless of the obstacle count.

Elements in action space 𝒜 include the joint angle velocity of the robot, defined as

(11) $$a≐\left[\dot{q}\right],$$

which is provided to the robot as the target velocity in action control.

#### 4.2.2. Neural Network Implementation

Similar to conventional reinforcement learning frameworks, the fast pathway utilizes parameterized neural networks (NNs) to approximate the stochastic policy π and the action-value functions *Q*. To mitigate value overestimation, the architecture employs twin current *Q*-networks parameterized by $\theta_{i}$ (i=1,2), where the minimum output of the two is selected for gradient computations. Furthermore, a target network technique is incorporated to stabilize critic training, wherein the target weights ${\bar{\theta }}_{i}$ are soft-updated based on the current weights $\theta_{i}$ with an update rate τ∈(0,1):

(12) $${\bar{\theta }}_{i}\leftarrow \tau \theta_{i}+\left(1-\tau \right){\bar{\theta }}_{i},\;i=1,2$$

Accounting for the actor, twin critics, and their corresponding target critics, the fast pathway comprises five parameterized NNs in total. Additionally, an experience replay pool 𝒟 and mini-batch random sampling are leveraged during the training phase to break data correlation and ensure sample independence. An ordinary uniform sampling strategy is adopted when drawing mini-batches from the replay buffer.

The actor network consists of an input layer, two fully connected hidden layers containing 256 units each, and two parallel output layers. The hidden layers employ the Rectified Linear Unit (ReLU) activation function. To parameterize the Gaussian policy distribution, the two parallel output layers yield the mean and standard deviation of the action distribution, utilizing the hyperbolic tangent (tanh) function and the exponential (exp) function as their respective activation functions. Symmetrically, each critic network adopts a comparable deep structure with two 256-unit hidden layers, mapping into a linear output layer that represents the scalar action value Q(s,a).

In this striatum-inspired model, the network optimization is driven by three coupled loss functions. Specifically, policy evaluation is performed by minimizing the critic loss $J_{Q}\left(\theta_{i}\right)$ defined in (7), which minimizes the soft Bellman residual to yield accurate action-value estimates. Concurrently, policy improvement is achieved by minimizing the actor loss $J_{\pi }\left(\phi \right)$ defined in (6), which steers the policy toward high-reward actions. Lastly, the temperature coefficient α is adaptively regulated via the entropy-related loss J(α) defined in (9) to dynamically balance exploration and exploitation.

To inherently mitigate state estimation uncertainties and observation noise originating from the upstream perception stack, stochastic state noise is explicitly injected into the policy inputs during the training phase. This domain-randomization mechanism forces the fast-pathway agent to learn a robust and generalized control policy capable of sustaining closed-loop safety under minor sensory fluctuations.

#### 4.2.3. Reward Design

Basically, the RL method enables the robot to learn reaching and obstacle avoidance skills through reward design. In the fast pathway, the scalar reward function is defined by the weighted sum of several terms as follows:

(13) $$r\left(s_{t},a_{t}\right)=r_{s}+w_{r}^{T}\left[\begin{array}{cccc} r_{g} & r_{a} & r_{o} & r_{p} \end{array}\right],$$

where $w_{r}$ is a weight vector and $r_{s}$ is a positive scalar representing survival reward.

(14) $$r_{g}=-{∥p_{ee}-p_{\mathrm{goal}}∥}^{2}$$

is the distance between the end-effector of the arm and the target point in the task space.

(15) $$r_{a}=-{∥a_{t}∥}^{2}$$

is the amplitude of the action.

(16) $$r_{o}=\left\{\begin{array}{cc} -r_{c}, & \mathrm{if}\;\mathrm{collided},\\ -{\left(\frac{d_{\mathrm{ref}}}{d_{o}+d_{\mathrm{ref}}}\right)}^{3}, & \mathrm{otherwise} \end{array}\right.$$

is relevant to $d_{o}$, the minimal distance between the robot and the obstacle in the task space. $d_{\mathrm{ref}}$ is a parameter controlling the shape of $R_{o}$.

(17) $$r_{p}=\left\{\begin{array}{cc} -1, & \mathrm{if}\;\mathrm{action}\;\mathrm{out}\;\mathrm{of}\;\mathrm{range},\\ 0, & \mathrm{otherwise} \end{array}\right.$$

is a penalty term for actions that exceed the constraints of the robot. In terms of obstacle collisions during training, the penalty $r_{c}$ is introduced when collision occurs, which can effectively promote learning obstacle avoidance strategies. As for the constraints introduced by robot dynamics, we use penalty terms $r_{p}$ instead of hard constraints to bring them into consideration.

The trade-off among competing objectives is regulated by the weight vector $w_{r}$, which is selected according to task priorities and reward magnitudes. Specifically, the goal-reaching reward $r_{g}$ and obstacle penalty $r_{o}$ are assigned larger weights to prioritize task completion and safety, while the action penalty $r_{a}$ serves as a regularization term that suppresses excessive control variations and promotes smoother motions. More advanced reward-learning approaches, such as inverse reinforcement learning and meta-reinforcement learning, are beyond the scope of this work, as the focus is on evaluating the proposed multi-pathway architecture rather than optimizing reward generation.

### 4.3. The Slow-Pathway Planning

The slow pathway aims to imitate the role of PFC in rational decision-making within the human brain, thereby facilitating long-term and holistic planning. Relevant neuroscientific evidence shows that the planning function of PFC is implemented through tree search, which correlates with the neural architecture and information processing mechanism within PFC [42]. Inspired by this notion, it is suggested that RRT-class sampling-based planning methods can be applied to mimic the planning function of the PFC, which similarly utilizes a searching tree structure to optimize decision costs and planning. Moreover, during the process of planning reconfiguration, the activity of PFC increases along with the planning demands of BFS way over future paths [43]. Hence, it is further suggested that the RT-RRT^*^ method, which also implements tree rewiring in a BFS manner, be employed to characterize the activity of PFC. As previously mentioned, the planning of the slow pathway adopts a hierarchical planning strategy, where initially, a path $\sigma_{p}$ is obtained through the RT-RRT^*^, and subsequently, trajectory planning methods are employed to derive the trajectory σ(ε).

#### 4.3.1. Planning State Space

In the planning process of the slow pathway, the planning state space 𝒳 is set as the joint space $𝒞\subseteq R^{m}$ of the robot. Although the joint space has a higher dimension compared to the task space, which adds complexity to the planning task, it significantly reduces the computational burden of inverse kinematics calculations and helps to avoid singular posture issues during robot motion. Therefore, the main steps of the slow pathway planning are conducted within the joint space.

In this situation, obstacles are transformed into the joint space to achieve collision detection. Note that ${𝒳}_{\mathrm{obs}}$ contains not only the mapping of the obstacle area to the joint space but also a set of robot configurations where collisions with obstacles occur, i.e.,

(18) $${𝒳}_{\mathrm{obs}}=\left\{x\in 𝒳|M\left(x\right)\cap 𝒪\neq \emptyset \right\},$$

where $M\left(x\right)$ and 𝒪 denote the representations of the robot under pose x and obstacle in the task space 𝒲.

#### 4.3.2. Sampling Strategy

The design of the sampling strategy is a critical factor influencing the efficiency of sampling-based planners. While some existing works integrate reinforcement learning into sampling-based planning—such as for computing the minimum time-to-reach (TTR) [44]—our approach leverages the output of an SAC actor to guide the sampling process within the slow pathway.

Specifically, the overall sampling distribution *f* in the state space 𝒳 is formulated as a hierarchical conditional distribution as follows:

(19) $$f\left(x_{a},ㄒ\right)=\left\{\begin{array}{cc} f_{\mathrm{fast}}\left(x_{a},ㄒ|\pi \right), & \mathrm{with}\;\mathrm{modulated}\;\mathrm{prob}.\;p_{f},\\ f_{r}\left(x_{a},ㄒ\right), & \mathrm{otherwise} \end{array}\right.$$

where $f_{\mathrm{fast}}\left(\cdot \right)$ denotes the sampling distribution modulated by the habitual motion decision-making model of the fast pathway. The selection probability $p_{f}$ is dynamically adjusted by emotional signals from the modulation module, the details of which are elaborated in Section 4.4.

If the fast-pathway guidance is not selected, the system falls back to a residual sampling strategy $f_{r}\left(\cdot \right)$, which is independent of the fast pathway and defined as

(20) $$f_{r}\left(x_{a},ㄒ\right)=\left\{\begin{array}{cc} I\left(x_{\mathrm{goal}}\right), & \mathrm{with}\;\mathrm{prob}.\;p_{g},\\ U\left(B\left(x_{a},x_{\mathrm{goal}},\sigma_{p}\right)\cap {𝒳}_{\mathrm{free}}\right), & \mathrm{with}\;\mathrm{prob}.\;p_{e},\\ U\left(𝒳\right), & \mathrm{otherwise}, \end{array}\right.$$

where $p_{g}$ and $p_{e}$ are probability parameters determining the selection of corresponding sampling strategies. The three branches of $f_{r}\left(\cdot \right)$ correspond to the following distinct strategic behaviors:

- Goal-Directed Biasing ($p_{g}$): I(·) represents the indicator function, where $I(x_{\mathrm{goal}})$ directly returns the target projection $x_{\mathrm{goal}}$. This explicitly guides the tree ㄒ toward the goal during the Steer phase.
- Informed Ellipsoid Sampling ($p_{e}$): U(·) denotes the uniform distribution. The planner samples within a hyperellipsoid B focused on $x_{a}$ and $x_{\mathrm{goal}}$. Its transverse and conjugate diameters are updated per decision period as
  (21) $$a_{e}=c\left(\sigma_{p}\right),\;b_{e}=\sqrt{c^{2}\left(\sigma_{p}\right)-d^{2}\left(x_{\mathrm{goal}},x_{a}\right)}.$$
  Such focused sampling accelerates the convergence of $\sigma_{p}$ while ensuring cost optimality [45]. If $x_{\mathrm{goal}}$ has not yet been discovered by ㄒ, this branch naturally degenerates into global uniform sampling.
- Global Exploration (otherwise): The planner samples uniformly from the entire free state space ${𝒳}_{\mathrm{free}}$ to maintain global exploration capabilities.

It is important to clarify that the primary purpose of the proposed sampling formulation is to investigate the influence of fast-pathway guided sampling. Specifically, the term $p_{f}$ is derived from the SAC-based fast-pathway policy and serves as the key modulation component that biases the sampling distribution toward behaviorally meaningful regions, thereby incorporating obstacle-avoidance tendencies learned from experience into the global planning process. As a result, the sampling bias is primarily governed by the learned policy rather than being strictly constrained by the environment. In contrast, $p_{g}$ and $p_{e}$ correspond to conventional goal-biased and informed sampling strategies commonly adopted in RRT-based planners, which are included to maintain baseline exploration efficiency in high-dimensional state spaces. Unlike obstacle-boundary biased sampling methods that primarily focus on navigating narrow passages, the proposed selective sampling strategy emphasizes the integration of learned behavioral priors from the fast pathway into global planning, enabling a more adaptive sampling distribution driven by decision-level experience.

Mathematically, the integrated multi-pathway framework preserves the probabilistic completeness of the underlying slow-pathway planner. Because the fast pathway only biases the sampling distribution rather than directly dictating the final motion commands, core mechanisms such as randomized tree expansion and rewiring remain intact. Furthermore, since Equations (19) and (20) maintain non-zero probabilities for unbiased sampling components, the planner retains its capacity to explore the state space outside the fast pathway’s guidance. This mitigates the risk of trapping the planner in local minima, ensuring that the fast pathway serves as an efficiency-enhancing heuristic without sacrificing the global exploration robustness of the slow pathway.

#### 4.3.3. Neighbor Searching Acceleration

In RT-RRT^*^, both the Nearest operation after sampling and the Near operation during the InsertNode and Rewire procedures require solving a nearest-neighbor search (NNS) problem, making NNS a major computational bottleneck of RRT-based algorithms. A straightforward implementation traverses the node set 𝒱 and identifies nodes within a distance threshold $\rho_{N}$, resulting in a computational complexity of O(N) that increases with the size of the search tree.

To improve search efficiency, the Hierarchical Navigable Small World (HNSW) algorithm [46] is adopted in the slow pathway. Inspired by skip-list structures, HNSW organizes nodes into multiple hierarchical graph layers and performs nearest-neighbor queries through coarse-to-fine navigation across these layers. This mechanism enables approximate nearest-neighbor retrieval with logarithmic average-case query complexity, reducing the search cost from O(N) to approximately O(logN). Compared with conventional methods such as KD-Tree [47] and Annoy [48], HNSW is particularly suitable for the continuously expanding RT-RRT^*^ tree because it supports efficient dynamic insertion of newly generated nodes while maintaining high query efficiency in high-dimensional joint spaces.

Accordingly, the Nearest operation is implemented as

(22) $$\text{Nearest}\left(ㄒ,x_{\mathrm{rand}}\right)=\mathrm{HNSWQuery}\left(ㄒ,x_{\mathrm{rand}},k=1\right),$$

where k=1 is used to retrieve the nearest node to $x_{\mathrm{rand}}$. Similarly, the Near operation is defined as

(23) $$\text{Near}\left(ㄒ,x_{\mathrm{new}}\right)=\left\{x\in \mathrm{HNSWQuery}\left(ㄒ,x_{\mathrm{new}},k=k_{N}\right)\;|\;d\left(x,x_{\mathrm{new}}\right)<\rho_{N}\right\},$$

where at most $k_{N}$ neighboring nodes are retrieved and filtered according to the neighborhood radius $\rho_{N}$. Here, $d\left(x_{1},x_{2}\right)={∥x_{1}-x_{2}∥}_{2}$ denotes the Euclidean distance in the robot joint space used for proximity evaluation during nearest-neighbor search and tree rewiring.

#### 4.3.4. Improvements in Path Planning

After each decision period, the RT-RRT^*^ algorithm computes a planning path $\sigma_{p}\left(ㄒ\right)$ for the current environment. If $x_{\mathrm{goal}}$ has already been found by ㄒ, $\sigma_{p}\left(ㄒ\right)$ returns the path corresponding to $c\left(x_{\mathrm{goal}}\right)$. Otherwise, $\sigma_{p}\left(ㄒ\right)$ returns the path corresponding to $c\left(x_{p}\right)$, where $x_{p}$ is determined by (24) as follows:

(24) $$x_{p}=\arg\;\min_{x\in V}\;\;c\left(x\right)+\lambda d\left(x,x_{\mathrm{goal}}\right)$$

where λ is a weight factor controlling the emphasis on both the planned path costs $c\left(x_{p}\right)$ and the distance from $x_{p}$ to the goal point.

**Figure 3:**
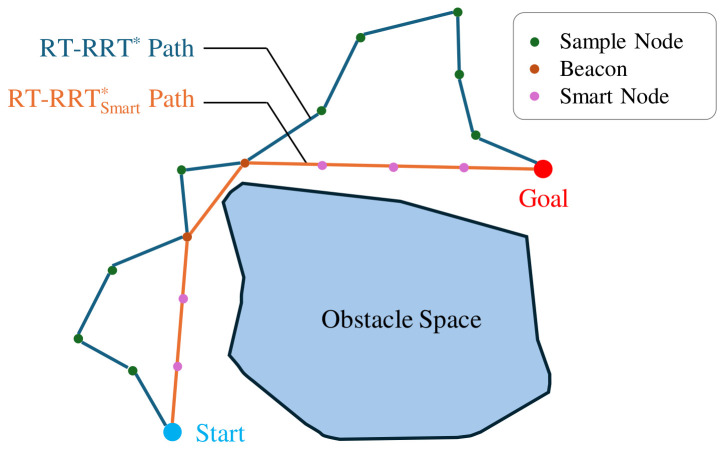
Geometric path optimization comparison between the standard RT-RRT^*^ and the proposed ${\mathrm{RT-RRT}}_{\mathrm{Smart}}^{*}$. The proposed framework deploys heuristic beacons and smart nodes, which dynamically rewires and smooths the local tree topology, yielding a shorter and more kinematically feasible path through the obstacle space. Abbreviations: RT-RRT^*^, Real-Time Rapidly-exploring Random Tree Star; ${\mathrm{RT-RRT}}_{\mathrm{Smart}}^{*}$, Real-Time Rapidly-exploring Random Tree Star with Smart technique.

RT-RRT^*^ inherits the probabilistic completeness and asymptotic convergence of the RRT^∗^ algorithm, providing it with an advantage in real-time decision-making for robot motion. However, its initial solutions are not favorable for robots, and the optimization convergence speed is relatively slow. In real-time planning tasks for robot motion decision-making, the generated redundant and rough paths are almost impractical for the robot to execute directly. Inspired by the RRT^∗^-Smart method [49], it is advised to introduce the Smart technique into the slow pathway to optimize the path, with the improved method called ${\mathrm{RT-RRT}}_{\mathrm{Smart}}^{*}$. Specifically, the Smart technique attempts to straighten the path polylines by connecting the latter path points to the former only if no collision is between them. The path points retained on the Smart path are called beacons. To eliminate collision risks against complex obstacle geometries during this pruning step, the system uniformly checks the line segment between the active beacon and subsequent candidate nodes to verify its clearance against the exact obstacle geometric boundaries. If a proposed shortcut segment is determined to be unsafe, the planner dynamically marks the last confirmed safe node as a new beacon and seamlessly resumes the forward visibility check from that position. This progressive backtracking strategy guarantees that the generated path remains highly optimized without risking physical collision. As shown in Figure 3, the path planned by ${\mathrm{RT-RRT}}_{\mathrm{Smart}}^{*}$ can significantly reduce redundancy compared to RT-RRT^*^, which is advantageous for trajectory planning and robot action execution.

It should be noted that the real-time planning ability of RT-RRT^*^ comes from the rewiring of the search tree in dynamic environments. Therefore, unlike RRT^∗^-Smart, after determining the beacons, the parents of the other original path nodes are not directly changed to the beacons, as this would disrupt the original search tree structure and cause conflicts with the rewiring steps. Instead, only the path output from the current ㄒ is modified. Meanwhile, considering that the positions of the robot and obstacles change during real-time planning, the original structure of ㄒ generated by RT-RRT^*^ is maintained without adjustment on the sampling strategy based on the beacons, which means the Smart technique is only applied when generating the path planning results.

On the other hand, the Smart path will cause the robot to deviate from the original path, which may affect the switching of the root node. Additionally, due to the removal of redundant nodes on the path, the interval between beacons may exceed the step size $\rho_{s}$. Therefore, after path planning, $x_{S}=Steer\left(x_{\mathrm{root}},x_{1}\right)$ is calculated as the new Smart node and is inserted into ㄒ if $x_{S}$ is not in ㄒ, which will serve as the next movement target of the agent and the next root of ㄒ. In an environment with static obstacles, this operation ensures the real-time availability of the search tree while maintaining the planning results of RT-RRT^*^. In this case, the probabilistic completeness condition of RRT^∗^ [38]

(25) $$\lim_{n\to \infty }P\left(𝒱\cap 𝒢\left(x_{\mathrm{goal}}\right)\neq \emptyset \right)=1$$

still holds, where $𝒢\left(x_{\mathrm{goal}}\right)$ represents the region where ㄒ is judged to reach $x_{\mathrm{goal}}$ in 𝒳.

#### 4.3.5. Trajectory Planning

The polyline paths formed by direct connections may not smooth enough and cause abrupt changes in robot action, making them unsuitable for the direct execution of the robot. Therefore, it is recommended that the trajectory be planned based on path points in joint space. Note that some works suggest using methods such as splines in path planning to obtain more detailed results and solve action sequences, while the computational cost of calculating splines between non-specific points is relatively high. In the slow pathway, it is suggested that the Time-Optimal Path Parameterization based on Reachability Analysis (TOPP-RA) method for spline trajectory can be used for planning trajectory and robot action after $\sigma_{p}\left(ㄒ\right)$ is obtained.

During each decision period, trajectory planning is carried out for the augmented path $\sigma_{pa}=\left\{x_{a}\right\}\cup \sigma_{p}\left(ㄒ\right)$, in which its elements are denoted by $\left\{x_{pa}^{i}\right\},\;\;i=0,1,\ldots ,N_{pa}$. The spline trajectory is defined as follows:

(26) $$\sigma_{i}\left(s\right)=h_{i0}+h_{i1}s+h_{i2}s^{2}+h_{i3}s^{3},s\in \left[s_{i-1},s_{i}\right],i=1,2,\ldots ,N_{pa}.$$

where $h_{il}$ are *m*-dimensional parameters, l=0,1,2,3.

(27) $$0=:s_{0},s_{1},\ldots ,s_{N_{pa}}:=1$$

is a monotonically increasing sequence defined on [0,1], determined by (28):

(28) $$s_{pa}^{i}=\left\{\begin{array}{cc} 0, & j=0,\\ \frac{\sum_{j=0}^{i-1}d\left(x_{pa}^{j-1},x_{pa}^{j}\right)}{\sum_{j=0}^{N_{pa}-1}d\left(x_{pa}^{j-1},x_{pa}^{j}\right)}, & 1\leq j\leq N_{pa}, \end{array}\right.$$

which facilitates the smooth motion of the robot along the path. Since the velocities at the beginning and end of the trajectory (${\dot{x}}_{a}$ and 0) are known, the spline parameters are uniquely determined by the interpolation conditions, the first- and second-order continuity conditions, and the boundary conditions. Specifically, these are expressed as:

(29) $$\left\{\begin{array}{c} \sigma_{i}\left(s_{i}\right)=x_{pa}^{i},i=0,1,2,\ldots ,N_{pa}\\ \frac{d\sigma_{i}}{ds}\left(s_{i}\right)=\frac{d\sigma_{i+1}}{ds}\left(s_{i}\right),i=1,2,\ldots ,N_{pa}-1\\ \frac{d^{2}\sigma_{i}}{ds^{2}}\left(s_{i}\right)=\frac{d^{2}\sigma_{i+1}}{ds^{2}}\left(s_{i}\right),i=1,2,\ldots ,N_{pa}-1\\ \frac{d}{ds}\sigma_{0}\left(0\right)=\frac{{\dot{x}}_{a}}{∥{\dot{x}}_{a}∥},\frac{d}{ds}\sigma_{0}\left(1\right)=0. \end{array}\right.$$

Thus, the complete shape of the trajectory σ(s) can be obtained.

Subsequently, the TOPP-RA method is utilized to compute the action sequence {u} for trajectory σ(s). TOPP-RA firstly recursively calculates the reachable and controllable sets of discrete positions along the path, and then greedily selects actions to construct an optimal state and action sequence, thereby achieving time-optimal trajectory planning along the specified path. Initially, let

(30) $$0=:s_{0},s_{1},\ldots ,s_{N_{ra}}:=1$$

denote another monotonically increasing linspace sequence defined on [0,1], where

(31) $$N_{ra}=k_{ra}\int_{0}^{1}∥\frac{d\sigma }{ds}∥ds$$

is proportional to the length of σ. $k_{ra}$ is a proportional parameter. Then, the controllable sets corresponding to $s_{i}$ can be computed through backward traversal:

(32) $$\begin{array}{c} K_{N_{ra}}=\left\{{\dot{s}}_{N_{ra}}^{2}\right\},\\ K_{i}=\left\{\Theta_{i}\left(K_{i+1}\right)\right\},i=N_{ra}-1,\ldots ,1,0, \end{array}$$

where $K_{i}$ represents the controllable set, comprising the states that can be reached by action sequences $\left\{u_{i+1:N_{ra}}\right\}$ to attain the state $x_{N_{ra}}$. $\Theta_{i}\left(K\right)$ denotes the one-step set, referring to the set of states that can be reached by a single action $u_{i}$ from the set *K*. Next, the optimization problems concerning {u} are solved through forward traversal via (33) and (34):

(33) $$\begin{array}{cc} \; & u_{i}^{*}=\max\;\;u\;\;\\ \; & s.t.\left\{\begin{array}{c} x_{i}^{*}+2\Delta_{i}u\in K_{i+1}\\ \left(u,x_{i}^{*}\right)\in \Omega_{i} \end{array}\right. \end{array}$$

(34) $$x_{i+1}^{*}=x_{i}^{*}+2\Delta_{i}u_{i}^{*}$$

where $\Delta_{i}=s_{i+1}-s_{i}$. $\Omega_{i}$ represents the feasible domain of the action–state pair $\left(u_{i},x_{i}\right)$, taking into account the robot’s kinematic and dynamic constraints.

Consequently, TOPP-RA returns an optimized action sequence {u} along the trajectory σ. At each control cycle, actions corresponding to the specific sampling timestamps are extracted from {u} and mapped onto the physical robot. Crucially, this architectural design decouples high-level kinematic output from low-level dynamic execution. Specifically, the upper-level motion planner strictly yields continuous reference joint positions, whereas the embedded low-level joint loop employs a compliant impedance controller. This configuration effectively absorbs transient chattering and circumvents the high-dimensional computational overhead traditionally required for online trajectory smoothing, thereby guaranteeing deterministic real-time responsiveness.

Although flexible-body effects are not explicitly incorporated into the rigid-body dynamic model, abrupt motion reversals are partially mitigated through the combined smooth trajectory generation of the slow pathway and the action-magnitude penalty embedded within the reinforcement learning reward function. These dual-layer mechanisms collectively encourage inherently smoother motion profiles and regularize aggressive control derivatives, which reduces the transient strains that could otherwise exacerbate unmodeled structural dynamics.

Finally, the process of the slow pathway is illustrated in Algorithm 2.
**Algorithm 2** The Slow Pathway: Hierarchical planning with ${\mathrm{RT-RRT}}_{\mathrm{Smart}}^{*}$ for path and TOPP-RA for trajectory  1: **Inputs:** $x_{\mathrm{init}},x_{a},{𝒳}_{\mathrm{obs}},x_{\mathrm{goal}}$, fast-pathway actor model π  2: Initialize Tree ㄒ and Queue $Q_{r},Q_{s}$  3: **repeat**  4:       Update $x_{a},{𝒳}_{\mathrm{obs}},x_{\mathrm{goal}}$  5:       **while** $t_{\mathrm{plan}}\geq 0$ **do**  6:             $x_{\mathrm{rand}}\leftarrow \text{Sample}\left(f\left(x_{a},ㄒ\right)\right)$  7:             $x_{\mathrm{nearest}}\leftarrow \text{Nearest}\left(ㄒ,x_{\mathrm{rand}}\right)$  8:             $x_{\mathrm{new}}\leftarrow \text{Steer}\left(x_{\mathrm{nearest}},x_{\mathrm{rand}}\right)$  9:             **if** $x_{\mathrm{new}}\notin {𝒳}_{\mathrm{obs}}$ **then**10:                   ${𝒳}_{\mathrm{near}}\leftarrow \text{Near}\left(ㄒ,x_{\mathrm{new}}\right)$11:                   $x_{\min}\leftarrow \text{ChooseParent}\left(x_{\mathrm{new}},{𝒳}_{\mathrm{near}}\right)$12:                   $ㄒ\leftarrow \text{InsertNode}(ㄒ,x_{\min},x_{\mathrm{new}})$13:                   ${\text{Rewire}}_{\mathrm{new}}\left(ㄒ,Q_{r},x_{\mathrm{new}}\right)$14:             **end if**15:             ${\text{Rewire}}_{\mathrm{root}}\left(ㄒ,Q_{s},x_{\mathrm{root}}\right)$16:       **end while**17:       $\sigma_{p}=\left\{x_{\mathrm{root}},x_{1},\ldots ,x_{k}\right\}\leftarrow \text{Smart-PathPlan}\left(ㄒ\right)$18:       $x_{S}\leftarrow \text{Steer}\left(x_{\mathrm{root}},x_{1}\right)$19:       **if** $x_{S}\notin ㄒ$ **then**20:            $ㄒ\leftarrow \text{InsertNode}(ㄒ,x_{\mathrm{root}},x_{S})$21:       **end if**22:       **if** $x_{a}$ is close to $x_{\mathrm{root}}$ **then**▹ Set New Root23:            $ㄒ\leftarrow \text{SetRoot}(x_{S})$24:       **end if**25:       $\sigma \leftarrow \text{TrajPlan}\left(x_{a},\sigma_{p}\right)$26:       $\left\{u\right\}\leftarrow \mathrm{TOPP-RA}\left({\dot{x}}_{a},\sigma \right)$27:       Agent move along $\sigma_{pa}$ with {u} for $\left\lfloor T_{d}/T_{c}\right\rfloor$ steps28: **until** $x_{a}$ reach $x_{\mathrm{goal}}$

### 4.4. Emotion-Based Multi-Pathway Modulation

In this section, a brain-inspired modulation mechanism is proposed to achieve smooth transitions between two types of motion decision-making. The modulation of the motion decision-making process is described as a finite state machine (FSM), with the fast pathway and slow pathway considered as two states within it. A simple approach is to set distance thresholds based on the kinematic characteristics of obstacles for state switching. However, this method leads to mutational state transition, which is inconsistent with the human decision-making modulation process.

Inspired by the role of emotional modulation in pathway transitions, a promising strategy is to draw inspiration from the functional role of the amygdala in emotional regulation to generate emotional signals based on the status of the robot and obstacles, including fear acquisition to facilitate habitual actions and fear extinction to facilitate the return to slow-pathway planning, thereby achieving smooth transitions between the two pathways.

**Figure 4:**
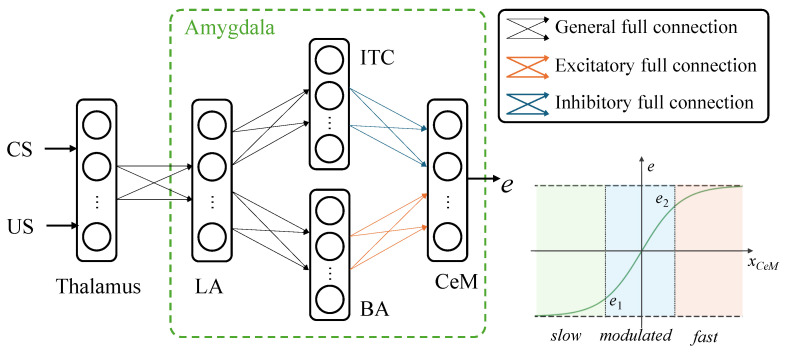
Architecture of the biologically inspired emotional modulation network EmoNet, whose connectivity is motivated by organizational principles reported in computational models of the amygdala, adapted from [50]. The network receives the conditioned stimulus (CS, denoting the state and obstacle observations) and the unconditioned stimulus (US, denoting collision indicators) via the thalamus. The inputs are processed through the lateral amygdala (LA), basal amygdala (BA), and intercalated cell clusters (ITCs), ultimately converging at the central amygdala (CeM) to output the continuous emotional signal *e*. The right panel illustrates the continuous emotional activation mapping. The emotional thresholds $e_{1}$ and $e_{2}$ divide the mode into three intervals: the planner-dominant slow pathway ($e<e_{1}$), the dual-pathway modulated transition ($e_{1}\leq e\leq e_{2}$), and the reactive fast pathway ($e>e_{2}$).

Specifically, drawing inspiration from the functional organization of the amygdala, a multilayer perceptron (MLP) neural network, EmoNet, is deployed, as illustrated in Figure 4. The network architecture incorporates connectivity patterns inspired by interactions among four functional amygdalar subregions reported in neuroscience studies: the lateral amygdala (LA), basal amygdala (BA), central amygdala (CeM), and intercalated cell clusters (ITCs) [50]. The weights of excitatory and inhibitory connections are constrained to positive and negative values, respectively. The inputs include $\left[q,\dot{q},p_{\mathrm{dyo}},{\dot{p}}_{\mathrm{dyo}}\right]$ as the conditioned stimulus (CS), and $I_{\mathrm{collision}}$ as the unconditioned stimulus (US), where $p_{\mathrm{dyo}}$ and ${\dot{p}}_{\mathrm{dyo}}$ are the position and velocity of the dynamic obstacle in the task space. $I_{\mathrm{collision}}$ is the indicator function of the collision event.

$e_{1}$ and $e_{2}$ are the emotional thresholds at which the slow pathway begins to be guided by the fast pathway and switches to the fast pathway, respectively. The operational priorities of the multi-pathway mechanism are dynamically and continuously modulated by the instantaneous emotional signal *e*, which is synthesized from the joint states of the robot, the spatial states of obstacles, and potential collision-related indicators. Higher emotional values typically correspond to a heightened potential threat from dynamic obstacles, subsequently leading to an increased activation priority of the fast pathway to ensure reactive safety. Such definition makes EmoNet suitable for dynamic obstacles that are being observed or suddenly appear.

The objective of EmoNet is to generate emotional signals *e* related to obstacles and collisions based on input information. Thus, the loss function is defined in (35) as follows:

(35) $$L\left(\theta_{e}\right)=\mathrm{MSE}\left(e,w_{e}^{T}\left[\begin{array}{cc} e_{d} & I_{collision} \end{array}\right]\right),$$

where $\theta_{e}$ is the weight of EmoNet. $e_{d}$ and $I_{collision}$ represent the impact of the distance to the dynamic obstacle $d_{\mathrm{dyo}}$ and the indicator function collision event on emotion, respectively. The weights are controlled by $w_{e}$. $e_{d}$ is defined as the decreasing function of $d_{\mathrm{dyo}}$ in (36) as follows:

(36) $$e_{d}=2\exp\left(-\zeta_{d}d_{\mathrm{dyo}}\right)-1,\;\zeta_{d}=-\frac{\log\frac{e_{2}-1}{2}}{d_{f}},$$

where $d_{f}$ is the reference distance to obstacles for $e_{2}$.

The output of EmoNet *e* represents the emotion signal caused by the approach of dynamic obstacles and collisions, which is utilized for two aspects of modulation in this approach. On one hand, *e* plays a role in the modulated probability $p_{f}$ of $f_{\mathrm{fast}}$ guided by the fast-pathway actor model in sampling function *f* of the slow pathway, as described in (37):

(37) $$p_{f}=\left\{\begin{array}{cc} 0, & e\leq e1,\\ p_{fm}\sqrt{\frac{e-e_{1}}{e_{2}-e_{1}}}, & e_{1}<e<e_{2}, \end{array}\right.$$

where $p_{fm}$ donates the maximum probability at $e=e_{2}$. $p_{f}$ increases as *e* increases, leading the robot to exhibit a propensity for planning along habitual action directions.

On the other hand, when $e_{1}<e<e_{2}$, it indicates that the robot may encounter potential threats posed by dynamic obstacles. Therefore, the robot should focus more attention on the current planning path. The design of $f_{\mathrm{fast}}$ is described in (38):

(38) $$f_{\mathrm{fast}}\left(x_{a},ㄒ|\pi \right)=I\left(x_{f}+\rho_{S}\frac{\pi \left(s_{pf}\right)}{∥\pi \left(s_{f}\right)∥}|\left(x_{f}∼U\left(\sigma \right)\right)\wedge \left(d\left(x_{a},x_{f}\right)<\rho_{f}\right)\right)$$

where I(·) denotes the indicator function. $x_{f}$ is a reference planning state in 𝒳 sampled along the current planned trajectory σ, with a distance not exceeding $\rho_{f}$ from $x_{a}$. $s_{f}$ represents the corresponding state of $x_{f}$, ${\dot{x}}_{f}$ on σ and the current environment. $f_{\mathrm{fast}}\left(\cdot \right)$ returns a sampled point at around $x_{f}$, moving along the direction of $\pi \left(s_{f}\right)$ with a step size of $\rho_{S}$. The sample radius

(39) $$\rho_{f}=\rho_{f0}\log\left(-\frac{e-e_{1}}{e_{2}-e_{1}}\right),e_{1}<e<e_{2}$$

decreases as *e* increases, indicating that as emotion heightens, $f_{\mathrm{fast}}$ tends to focus on planning points closer to $x_{a}$ along σ, which enables the robot to pay more attention to the surrounding conditions at the current position after obstacle avoidance actions, thus accelerating the optimization and adjustment of local planning. In (39), $\rho_{f0}$ is a custom parameter.

With the above modulation mechanism, the motion decision-making type can be classified into three scenarios: when $e<e_{1}$, the robot conducts fully slow-pathway planning independent of the fast pathway; when $e_{1}<e<e_{2}$, the robot engages in slow-pathway planning guided by the habitual actions; and when $e>e_{2}$, the robot executes habitual actions directly under the fast pathway. Considering the continuity of emotional signals, *e* is soft-updated in practical deployment, as depicted in (40):

(40) $$e_{t+1}=\tau_{e}e_{t}+\left(1-\tau_{e}\right)e,$$

where $\tau_{e}$ is the coefficient of emotion soft-update.

The proposed EmoNet is intended as a functional abstraction of emotional modulation. It should be noted that EmoNet serves as a biologically inspired engineering abstraction that incorporates several qualitative organizational principles reported in neuroscience studies to facilitate pathway modulation in robotic motion decision-making. Within this framework, the emotional thresholds $e_{1}$ and $e_{2}$ serve as modulation parameters that govern the transition between deliberative planning and reactive behaviors, while the soft-update coefficient $\tau_{e}$ controls the temporal smoothness of pathway switching. Parameters such as $e_{1}$, $e_{2}$, and $\tau_{e}$ are selected empirically to achieve stable modulation behavior in the considered robotic tasks. Specifically, excessively low values of $e_{1}$ and $e_{2}$ may cause frequent activation of the fast pathway in response to minor environmental fluctuations, whereas excessively high thresholds may delay the engagement of reactive avoidance behaviors under collision risk. Small values of $\tau_{e}$ may slow the adaptation of the modulation state to environmental changes, while large values may lead to more abrupt pathway transitions. Moderate parameter values help maintain a balanced interaction between reactive safety and deliberative planning.

It should be noted that the proposed framework does not directly combine motion commands generated by the two pathways. Instead, the modulation mechanism selects the pathway with higher priority according to the current environmental conditions. Consequently, only one pathway provides the motion command to the controller at each decision instant, avoiding potential conflicts between simultaneously generated motion vectors.

The framework mitigates undesirable control oscillations through a coordinated planning-and-control hierarchy. First, the transition between the slow and fast pathways is formulated as a continuous probability modulation via the emotional valence *e*, rather than an abrupt binary hard-switch; under low-threat conditions, the fast pathway merely injects intuitive guidance into the global sampling process. Second, when *e* exceeds the threshold and activates intensive reflexive steering, substantial changes in the control commands are both biologically natural and physically necessary to guarantee high-speed obstacle avoidance.

Overall, the operational procedure of the integrated multi-pathway framework is detailed in Algorithm 3. Since the planning process is performed in a receding-horizon manner, updated dynamic obstacle states and environmental information are continuously incorporated into each sequential iteration. If the slow pathway does not generate a newly updated, collision-free motion within the current control cycle, the robot safely continues executing the previously validated safe planning result while simultaneously initiating a replanning loop in the subsequent cycle.
**Algorithm 3** Multi-Pathway Motion Decision-Making  1: **Inputs:** 
$x_{\mathrm{init}},x_{a},s_{t},{𝒳}_{\mathrm{obs}},x_{\mathrm{goal}},\pi ,\;\mathrm{and}\;\mathrm{EmoNet}$  2: Initialize ㄒ, $Q_{r},Q_{s}$ for Slow-Pathway, count←0  3: **repeat**  4:       Update $s_{t},{𝒳}_{\mathrm{obs}}$  5:       $e_{t}\leftarrow \mathrm{EmoNet}\left(\mathrm{CS},\mathrm{US}|s_{t}\right)$  6:       **if** $e_{t}>e_{2}$ **then**  7:             count←0  8:             $\left\{u\right\}\leftarrow \left\{\pi \left(s_{t}\right)\right\}$▹ Fast Pathway  9:       **else**10:             **if** $\mathrm{count}\geq T_{d}/T_{c}$ **then**11:                   count←012:                   **if** $e_{t}<e_{1}$ **then**13:                          $\left\{u\right\}\leftarrow \text{Slow-Pathway}\left(x_{a},x_{\mathrm{goal}},{𝒳}_{\mathrm{obs}}\right)$14:                   **else**15:                          $p_{f},\rho_{f}\leftarrow \text{Modulation}\left(e_{t}\right)$16:                          $\left\{u\right\}\leftarrow \text{Slow-Pathway}\left(x_{a},x_{\mathrm{goal}},{𝒳}_{\mathrm{obs}}|p_{f},\rho_{f}\right)$17:                   **end if**18:             **end if**19:             count←count+120:       **end if**21:       Agent move according to {u} for time $T_{c}$22: **until** $x_{a}$ reach $x_{\mathrm{goal}}$


## 5. Experiment

### 5.1. Experimental Setup

**Figure 5:**
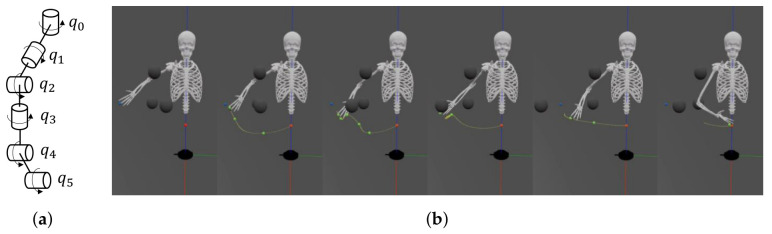
Experimental setup and physical simulation of the dynamic reaching task. (**a**) Kinematic modeling of the 6-DoF musculoskeletal humanoid manipulator, corresponding functionally to the spatial articulations of the shoulder, elbow, and wrist. (**b**) Time-lapse visualization of the dynamic obstacle avoidance process in simulation. The curve and green dots represent the planned spatial path and intermediate waypoints. The blue and red dot indicates the start and target position, respectively. The black spheres denote the dynamic obstacles. The manipulator coordinates multi-pathway decisions to track the planned spatial path and reach the goal, while simultaneously adopting reactive action to avoid dynamic obstacles in the workspace.

Referencing the upper-limb model structure in OpenSim [51] (an open-source platform used for simulating human movement), a 6-DOF humanoid arm is constructed to evaluate the multi-pathway method, which comprises a 3-DOF shoulder joint, a 2-DOF elbow joint, and an 1-DOF wrist joint, as illustrated in Figure 5a. The kinematic configuration of the 6-DOF humanoid arm is established with a standardized rigid-body tree topology parsed from a Unified Robot Description Format (URDF) description file. The geometric topology, link dimensions, and joint degrees of freedom are configured to approximately reproduce the kinematic characteristics of a human upper limb. Joint position and velocity limits are configured according to human-arm-inspired kinematic constraints and enforced as hard boundaries during simulation. The motion planner incorporates the geometric angular bounds and kinematic velocity limits specified in the URDF as explicit hard boundaries during simulation.

As shown in Figure 5b, the arm is set to carry out goal-directed tasks while avoiding collisions with obstacles. The end-effector is required to reach the target point position without specifying its posture. Such redundancy in robot degrees of freedom relative to the task provides the robot with additional planning flexibility. Considering that $x_{\mathrm{goal}}$ corresponds to a lower-dimensional area in joint space under this circumstance, an inverse kinematics solver with a masking technique is utilized to calculate the distance between the current position and the goal area in the joint space to assess proximity to the endpoint. The masking-based inverse kinematics solver is used for goal-distance evaluation and does not participate in motion generation or redundancy resolution optimization. Since motion planning and action generation are performed directly in the joint space, the framework does not explicitly address Jacobian singularities associated with operational-space control.

Static obstacles are randomly set within a certain range near the initial pose of the robot, which ensures reasonable interference to the arm and experimental consistency. In the slow-pathway planning process, collisions are judged by uniformly interpolating intermediate configurations along each candidate line segment with a maximum joint-space spacing of $\Delta q_{\mathrm{check}}=0.02$ rad between adjacent samples. Regions within 0.05 m of obstacles in the task space are considered obstacle zones. The metric *d* is set as the Euclidean distance.

During each simulation cycle, the framework utilizes explicit geometric collision queries performed directly on the exact rigid-body mesh geometries parsed from the URDF model. The simulation environment leverages internal hierarchical bounding volume trees to compute the three-dimensional Euclidean distances between the robot links and obstacle boundaries in real time. Simulation tests are conducted in the Swift simulation environment using the Robotics Toolbox for Python (Ver. 1.0.3) [52], which offers efficient collision detection and obstacle distance calculation capabilities. In the slow-pathway planning process, the collisions are judged by uniformly sampling along the line segment to be checked. The control period of the humanoid arm in simulation is $T_{c}=0.05$ s, while the decision period of the slow pathway is $T_{d}=0.2\;s$, spanning four control periods.

All experiments are conducted in simulation on a computer equipped with an Intel Core i7-11700 processor and 32 GB RAM. Consequently, discrepancies between planned trajectories and physically executed motions arising from actuator dynamics, transmission effects, sensor noise, or controller imperfections are not explicitly evaluated. Assessing such execution errors on a physical robotic platform remains an important direction for future work.

### 5.2. Training of Fast-Pathway Action Model

Firstly, the action model for the fast pathway is trained in a dynamic obstacle-avoidance environment. Three randomly moving obstacles are introduced to promote exploration of the state space. The obstacles move around the manipulator with periodically changing velocities and occasionally approach the wrist or elbow to encourage the learning of reactive avoidance behaviors. When an obstacle collides with the robot or reaches the boundary of its motion range, its kinematic state is reset. Training updates are performed once the replay buffer contains sufficient samples for mini-batch optimization, where network parameters are updated after each interaction step when the memory size exceeds the batch size threshold.

The training parameters of the fast pathway are summarized in Table 1. These parameters are selected based on preliminary experiments to provide stable learning performance in dynamic obstacle environments. In particular, the discount factor is set to γ=0.9. Rather than being optimized through a dedicated search procedure, this value is adopted following common practice in SAC-based robotic control tasks. A relatively moderate discount factor allows future rewards associated with task completion to remain influential while increasing the contribution of immediate penalties related to obstacle proximity and potential collisions. This configuration is consistent with the role of the fast pathway, whose primary objective is to generate rapid obstacle-avoidance actions rather than independently accomplish long-horizon motion planning.

The safety margin around the arm is enforced through two complementary mechanisms. First, the obstacle-related reward in Equation (16) continuously penalizes proximity to obstacles according to the reference robot–obstacle distance $d_{\mathrm{ref}}$, thereby encouraging the agent to maintain a non-zero clearance during motion. Second, a conservative collision threshold of $d_{\min}=0.05\;m$ is adopted during training. Any obstacle entering this distance is treated as a collision event and triggers the collision penalty. This threshold effectively introduces an additional safety buffer around the robot links, encouraging obstacle-avoidance behavior before physical contact occurs.

Exploration is achieved through the stochastic policy of SAC rather than externally injected noise. During training, actions are sampled from the learned policy distribution, while entropy regularization encourages continuous exploration of the state–action space. Therefore, no additional exploration-noise scheduling strategy is introduced.

**Figure 6:**
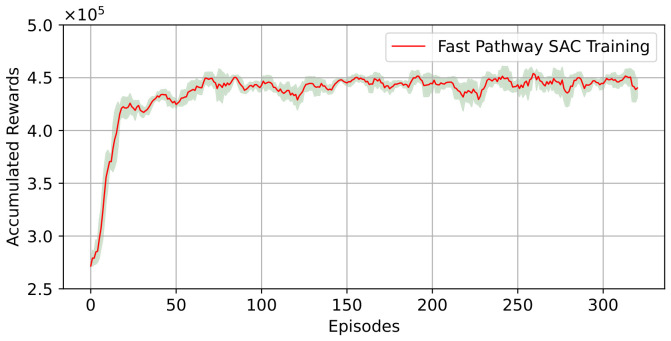
Accumulated reward of the fast-pathway SAC policy. The red solid line represents the mean cumulative reward, and the light green shaded region denotes the standard deviation.

Figure 6 illustrates the accumulated reward of the trained action model in training. The values correspond to the mean reward of the last five training rounds, with the shaded area representing the standard deviation. The initial rise and subsequent convergence in reward indicate the effectiveness of the training method. The trained action model enables the arm to reach and stabilize near the target point while actively dodging obstacles to maximize rewards. Each training epoch consists of 1000 interaction steps. Under the parameter settings in Table 1, the SAC agent typically reaches a stable accumulataed reward plateau after approximately 100 training epochs. To further improve robustness and ensure convergence, training is continued for a total of 310 epochs. The total wall-clock training time for the entire process is approximately 7 h.

**Figure 7:**
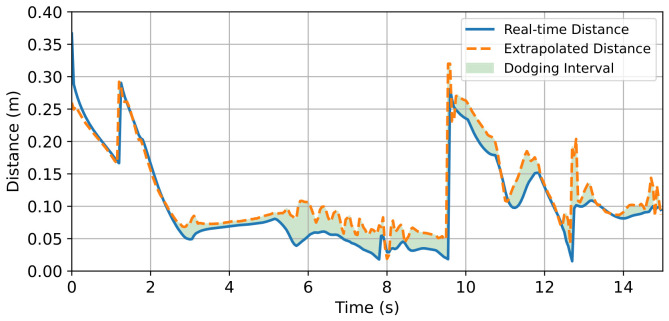
Real-time distance $d_{o}$ and extrapolated distance ${\tilde{d}}_{o}$ during dynamic obstacle avoidance with fast-pathway action model.

To validate the obstacle avoidance effectiveness of the fast pathway, the variation in $d_{o}$ is further investigated in the same environment. In addition, let ${\tilde{d}}_{o}$ represent the extrapolated distance, calculated by assuming that the arm continues executing the current action for another five control cycles, while keeping the obstacle positions fixed. Note that the extrapolated distance is a hypothetical measure assuming static obstacle positions and unchanging action signals that is used to evaluate the immediate tendency of the current action. Figure 7 shows the variation in both $d_{o}$ and ${\tilde{d}}_{o}$. The sudden fluctuations in the curves are caused by the re-deployment of obstacles upon reaching the boundaries of the environment. It can be observed that when $d_{o}$ decreases, the extrapolated distance ${\tilde{d}}_{o}$ is often larger than the current $d_{o}$, indicating that the actor is generating actions aimed at increasing the separation from obstacles, which reflects effective short-term obstacle-avoidance behavior.

Related research in behavioral studies has found that individuals tend to maneuver around obstacles and maintain the minimum distance between the actuator and obstacles in human obstacle-avoidance tasks [53]. As depicted in Figure 7, it can be observed that when obstacles move towards the arm (e.g., in 3–9 s), it tends to maintain a gap from the obstacle, which to some extent indicates that the employed method enables the robot to acquire human-like motion decision-making and avoidance abilities in obstacle environments.

In addition, The EmoNet of the modulation module is also pre-trained before the joint implementation of the complete framework. Specifically, supervised training is conducted together with the fast-pathway action model in the same training environment described in Section 5.2. Training is considered converged when the loss value stabilizes at a sufficiently low level over consecutive iterations. The transferability of EmoNet across different environments is primarily related to its input formulation. Specifically, EmoNet relies on local robot states, collision-related indicators, and the relative positions and velocities of nearby dynamic obstacles, rather than global environmental topologies or a fixed number of obstacles. As a result, the learned emotional modulation mechanism is less dependent on a specific environment configuration and remains applicable to scenarios with different obstacle layouts and motion patterns. In the following experiments, the trained actor model and EmoNet are utilized for the fast pathway and the modulation module, respectively.

### 5.3. Evaluation of Algorithm Performance and Human-like Characteristics

Next, the effectiveness of the multi-pathway approach is evaluated through goal-oriented tasks in the environment with both static and dynamic obstacles. The experimental setup consists of two stationary obstacles and one periodically moving obstacle, oscillating between points [0.3, −0.15, 0.5]^T^ and [0.3, −0.65, 0.5]^T^ near the target. The arm is asked to move from initial posture $q_{\mathrm{init}}=\;$[0.838, 1.082, 1.553, −1.187, 1.571, 0]^T^ to the target point of end-effector $p_{\mathrm{goal}}=\;$[0.4, 0, 0.5]^T^.

The details of the parameters of the slow pathway as well as the modulation module are listed in Table 2. It can be verified that the neighbor radius $\rho_{N}$ for rewiring satisfies the asymptotic convergence condition of RRT^∗^ [38], as follows:

(41) $$\rho_{N}>2{\left(1+\frac{1}{m}\right)}^{\frac{1}{m}}{\left(\frac{\mu \left({𝒳}_{\mathrm{free}}\right)}{\xi_{m}}\right)}^{\frac{1}{m}}$$

where *m* is the dimension of the workspace, $\mu \left({𝒳}_{\mathrm{free}}\right)$ is the Lebesgue measure of ${𝒳}_{\mathrm{free}}$, and $\xi_{m}$ is the volume of the unit ball in the workspace.

**Figure 8:**
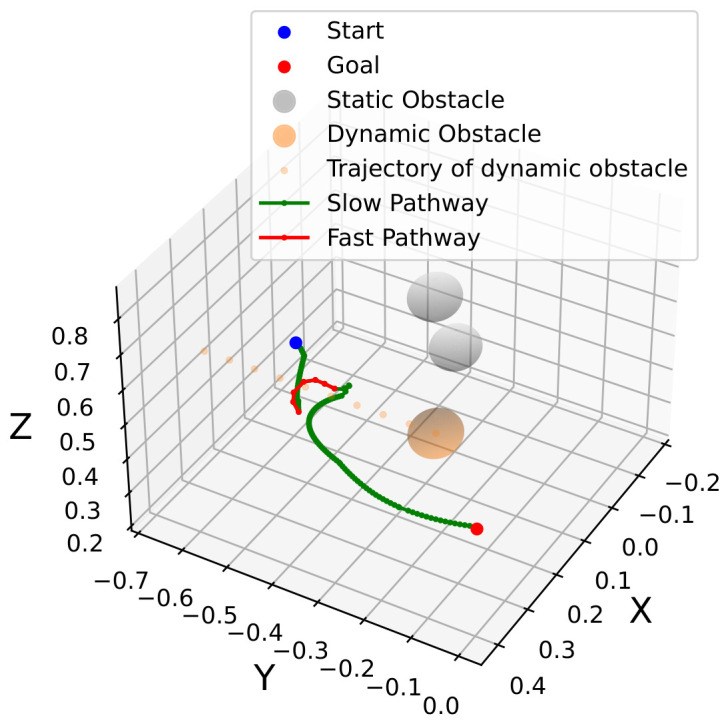
End-effector spatial trajectory with multi-pathway method.

**Figure 9:**
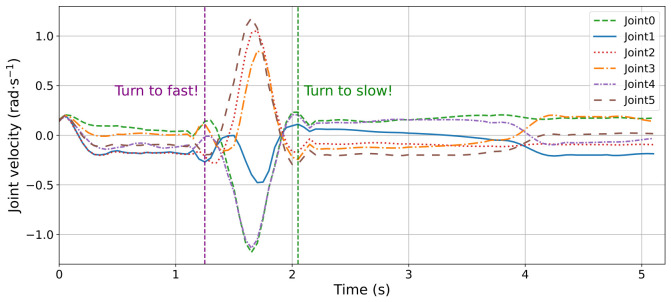
Joint velocity profiles of the 6-DoF manipulator during the dynamic obstacle avoidance process. The region bounded by dashed lines indicates the transient intervention of the fast pathway.

Figure 8 and Figure 9 demonstrate the trajectory of the end-effector and joint velocity, respectively. The arm successfully finds a path to reach the goal through slow-pathway planning. Meanwhile, one instance of fast-pathway habitual avoidance occurs during the process, enabling the robot to circumvent the approaching dynamic obstacle and subsequently resume slow-pathway planning toward the target, ultimately accomplishing the task. The fast pathway is activated for 17.5% of the task duration, indicating that the proposed framework operates predominantly in the deliberative planning mode, while reactive avoidance is invoked only during short periods when immediate collision threats are detected.

The proposed framework adopts a dual-rate architecture, where SAC policy evaluation and EmoNet inference are performed at every control cycle, while ${\mathrm{RT-RRT}}_{\mathrm{Smart}}^{*}$ planning is executed at a slower decision cycle. Since the fast pathway and EmoNet are implemented as lightweight MLPs, their inference times are typically below 1ms and contribute only a small fraction of the overall computational load. The primary computational cost arises from RT-RRT^*^ tree expansion and collision checking in the slow pathway. Although the planning cost increases with the dimensionality of the configuration space and the number of sampled nodes, HNSW-based neighbor searching alleviates part of this burden. Furthermore, the slow pathway only needs to generate a short-horizon motion segment for subsequent control cycles rather than a fully converged global solution within each decision period. Consequently, the running speed of the framework satisfies the real-time requirements of the task. While the slow pathway alone may be insufficient to handle high-frequency environmental perturbations because of its search overhead, the fast pathway provides instantaneous reactive actions when environmental changes occur faster than the planner can complete an update cycle, thereby enhancing closed-loop reactive safety.

To further evaluate the real-time performance of the proposed framework, the computational latency of the slow pathway is profiled under different NNS methods and tree sizes, as summarized in Table 3. The runtime is decomposed into nearest-neighbor query, collision checking, TOPP-RA trajectory optimization, and neural network inference. The results show that HNSW consistently achieves the shortest overall planning cycle among the evaluated methods. As the tree size increases from 1000 to 4000 nodes, the total planning time remains below the 200 ms decision interval, demonstrating the feasibility of real-time execution. In contrast, the computational cost of brute-force NNS increases rapidly with tree size, while KD-Tree exhibits intermediate performance. It is worth noting that the collision checking time also differs among the NNS methods. Since collision validation is performed for the candidate parent and rewiring nodes returned by the neighborhood search, approximate nearest-neighbor retrieval in HNSW leads to a smaller candidate set than exact search methods, thereby reducing the number of collision checking operations during tree expansion. Therefore, the observed reduction in collision checking time is a consequence of the interaction between neighborhood retrieval and the planning process.

**Figure 10:**
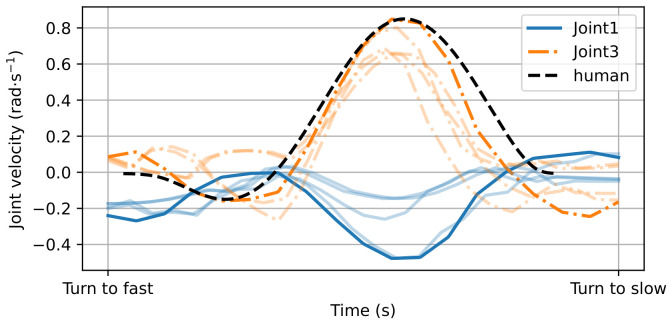
Output trajectory of Joint 1 and Joint 3 during the transition from the slow pathway to the fast pathway, compared with the human behavior result. The lighter-colored lines indicate the trajectory distribution sampled from multiple experiments.

Moreover, the motion trajectory characteristics of the arm under the proposed multi-pathway approach are analyzed and compared with those of the human arm in obstacle avoidance scenarios. Figure 10 illustrates the velocity profiles of two representative joints, corresponding to the robot’s shoulder (Joint 1) and elbow (Joint 3). The lighter-colored curves represent the results from multiple independent experiments. The segments of the robot’s velocity trajectories are also compared with the human joint velocity data in similar tasks, as reported in [54]. The third part in the right panel of Figure 2 from [54] is reproduced in Figure 10 for direct comparison.

It can be observed that, firstly, the joint velocities during the avoidance process exhibit bell-shaped profiles, which are qualitatively consistent with characteristic patterns reported in human arm movements [55,56]. Secondly, the joint velocities demonstrate a trend of deceleration followed by avoidance behavior. This phenomenon is consistent with previous observations and modeling results of human obstacle avoidance [57], reflecting two commonly reported features of human obstacle-avoidance movements: deceleration and obstacle circumvention [58]. Finally, the overall temporal evolution of the velocity profiles is consistent with those reported in human obstacle-avoidance tasks. To complement the qualitative comparison, the normalized joint velocity profiles are quantitatively compared with the human reference using the Pearson correlation coefficient. After aligning the joint motion directions, the proposed method achieved average correlation coefficients of 0.88±0.04 for Joint 1 and 0.85±0.03 for Joint 3, indicating a high degree of similarity in the temporal evolution of the velocity profiles. These results suggest that the proposed multi-pathway framework reproduces several motion characteristics reported in human obstacle-avoidance studies, particularly the bell-shaped velocity profiles and the deceleration-before-avoidance pattern. However, the proposed framework is not intended to reproduce human motor behavior in a biomechanical sense. Instead, it is designed to capture human-inspired decision-making characteristics through the coordination between deliberative planning and rapid reactive avoidance. Accordingly, the similarity to human motion should be interpreted within the scope of these specific motion characteristics.

These observations suggest that the proposed multi-pathway approach can generate motion characteristics that are qualitatively consistent with certain aspects of human obstacle-avoidance behavior. It should be noted, however, that the present comparison is qualitative and does not constitute a formal biomechanical validation. Quantitative evaluations based on human-subject datasets and dedicated similarity metrics remain an important future direction.

The proposed framework is formulated in joint space and relies primarily on robot states, obstacle distances, and target information rather than morphology-specific kinematic features. Consequently, the overall architecture is expected to be applicable to manipulators with different kinematic structures, including higher-dimensional systems such as dual-arm platforms, after retraining the fast pathway and updating the corresponding robot model. However, as the dimensionality of the configuration space increases, the computational cost of sampling-based planning, nearest-neighbor retrieval, and collision checking is expected to increase due to the enlarged search space and denser tree expansion. Although the underlying multi-pathway architecture remains unchanged, maintaining the same real-time performance on higher-dimensional manipulators may require additional computational resources, additional parameter tuning, or further optimization of the planning algorithm. Nevertheless, the present study evaluates the framework only on a 6-DoF humanoid arm. A systematic investigation of computational scalability, cross-morphology transfer, and validation on higher-dimensional robotic platforms remains an important direction for future work.

### 5.4. Evaluation in Complex Environment

**Figure 11:**
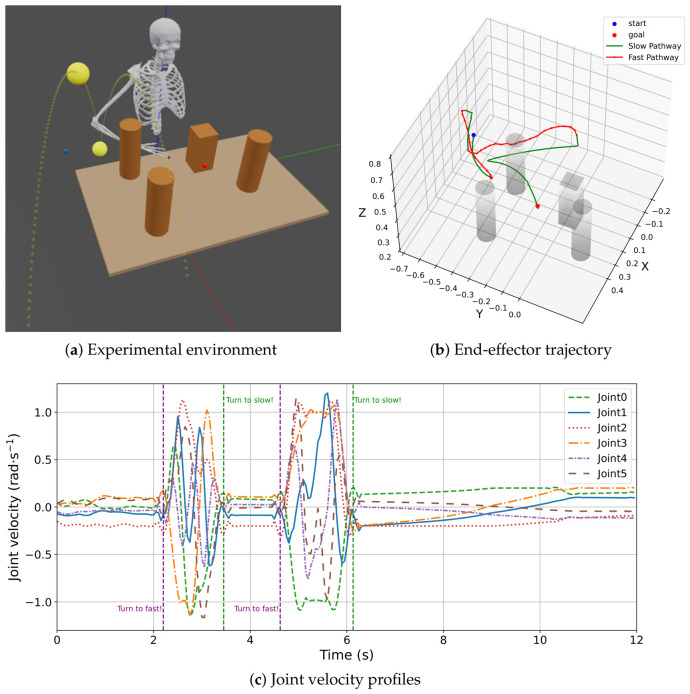
Performance evaluation of the proposed multi-pathway framework in a complex scenario with densely distributed obstacles. (**a**) The simulation setup, featuring a mixture of uncooperative dynamic spheres and static geometric constraints. The yellow spheres and their dotted trajectories indicate dynamic obstacles, while the blue and red dots represent the start and goal positions, respectively. (**b**) The 3D spatial trajectory of the end-effector, demonstrating successful navigation and continuous collision avoidance. (**c**) Joint velocity outputs ($q_{0}$–$q_{5}$) corresponding to the dynamic evasion process, highlighting the reactive actions triggered by the fast pathway in emergency.

To further evaluate the adaptability of the proposed method to complex dynamics, another environment with multiple dynamic obstacles is constructed, as illustrated in Figure 11a. This scenario simulates a situation in which the robot needs to deal with suddenly emerging dynamic obstacles. Specifically, the arm is required to reach a target through several static obstacles placed on a table and simultaneously avoid two consecutively thrown dynamic balls. The parameters of the method are kept the same as those in Section 5.3 for comparability. The resulting end-effector trajectory and joint velocity profiles are presented in Figure 11b and Figure 11c, respectively. As observed, the arm executes two avoidance maneuvers to successfully evade the oncoming balls. In contrast to the environment in Section 5.3, where the dynamic obstacle follows predictable motions, this experiment introduces sudden disturbances with high uncertainty, thereby prompting the robot to respond more rapidly to ensure safe and effective task completion.

In addition, performance metrics are evaluated through repeated trials. To ensure the existence of feasible solutions, the degree of randomness in the initial conditions, including $q_{\mathrm{init}}$ and $p_{\mathrm{goal}}$, is constrained within a range of ±0.1. Across multiple runs, the proposed algorithm achieves a success rate of 85%. In the successful cases, the average time for the manipulator to reach the target is 12.67±1.83 s, with an average path length of 3.16±0.42 m. The primary cause of failure in the remaining trials was that the first avoidance maneuver occasionally led the arm into a configuration where subsequent evasion of the second dynamic obstacle became difficult.

It should be noted that the dynamic obstacles considered in the current experiments mainly follow representative motion patterns with relatively structured trajectories. The proposed framework does not explicitly assume a predefined obstacle motion model, as both the emotion modulation mechanism and the motion decision-making process are driven by real-time observations. This design may allow the framework to respond to obstacle motions that are irregular, non-linear, or difficult to predict. Nevertheless, the performance under highly adversarial or chaotic obstacle behaviors was not systematically evaluated in the present study. Obstacle motions with extremely high accelerations or velocities may challenge the physical actuation limits of the robot and consequently affect avoidance performance.

### 5.5. Comparative Experiment

To investigate the contribution of the proposed brain-inspired multi-pathway mechanism, comparative experiments are conducted against sampling-based planning methods and reinforcement learning baselines in reaching tasks involving both static and dynamic obstacles, similar to those in Section 5.3 with static and dynamic obstacles.

The selected baselines are intended to evaluate the individual capabilities of the slow and fast pathways, thereby enabling a more direct assessment of the contribution introduced by the proposed multi-pathway coordination mechanism. Although hierarchical decision-making approaches, such as option-based and hierarchical reinforcement learning frameworks, are also relevant to complex robotic decision-making problems, their performance is often influenced by task decomposition strategies and high-level abstraction designs. Therefore, the present study focuses on representative planning-based and reinforcement learning baselines to provide a controlled evaluation of the proposed brain-inspired multi-pathway architecture.

**Figure 12:**
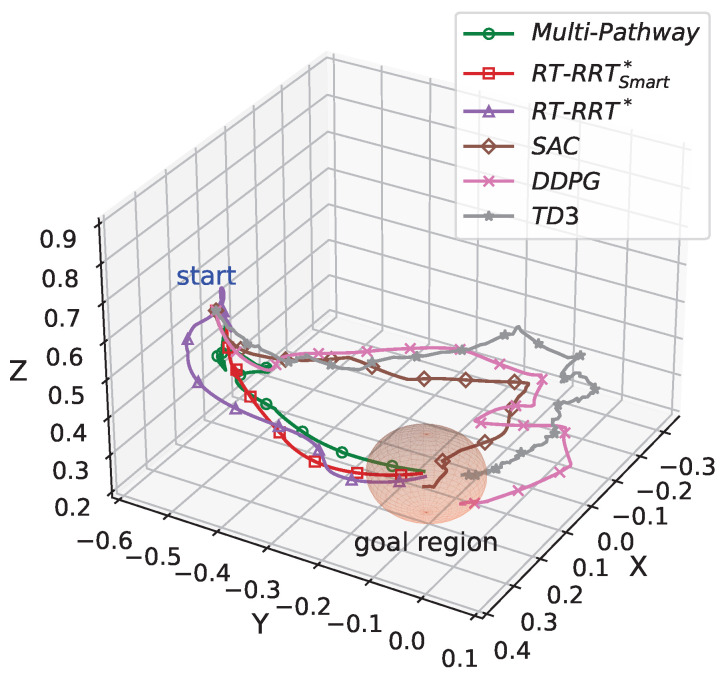
Trajectory of end-effector under different methods in obstacle environment.

Figure 12 illustrates the end-effector trajectories under various algorithms, and the corresponding joint velocity profiles are provided in Appendix B. Qualitatively, planning-based methods (Multi-Pathway, ${\mathrm{RT-RRT}}_{\mathrm{Smart}}^{*}$, and RT-RRT^*^) generate more direct and less redundant trajectories than RL methods under identical kinematic and dynamic constraints, enabling the arm to traverse the obstacle region and reach its goal with relatively direct paths. Notably, the integrating of the Smart optimization mechanism yields more concise paths for Multi-Pathway and ${\mathrm{RT-RRT}}_{\mathrm{Smart}}^{*}$ during the initial planning phase when the search tree has not fully explored the state space. Furthermore, when encountering dynamic obstacles, the proposed multi-pathway method exhibits human-like evasion maneuvers. In contrast, the ${\mathrm{RT-RRT}}_{\mathrm{Smart}}^{*}$ method induces behavioral hesitation and local trajectory adjustments; the robot only resumes its original trajectory after the obstacles clear. The results sufficiently validate the obstacle-avoidance capability and planning efficiency of the proposed framework.

Conversely, RL methods (SAC, TD3, and DDPG) attempt to navigate around obstacle regions via roundabout paths, resulting in comparatively rough trajectories. While the SAC trajectory remains relatively stable, DDPG exhibits pronounced proactive avoidance maneuvers near the target zone due to approaching dynamic obstacles. These behaviors indicate that although RL allows the robot to acquire target-reaching and flexible avoidance skills, it remains challenging to learn smooth obstacle-traversal strategies solely through non-linear obstacle rewards (16).

To ensure statistical reliability and experimental consistency, multiple trials are conducted for each method. Several quantitative metrics are evaluated, including the average success rate, average time-to-goal, average path length, and jerk cost. A trial is considered successful if the end-effector reaches the target region, defined as a sphere with a radius of 0.1m, within 10s without colliding with any obstacles. In addition, motion smoothness is assessed with the jerk cost, which evaluates the localized motion smoothness by integrating the joint jerk throughout the entire task horizon. It is mathematically formulated as follows:

(42) $$J_{\mathrm{Jerk}}=\int_{0}^{T}{∥\dddot{q}\left(t\right)∥}^{2}dt$$

where $\dddot{q}\left(t\right)$ denotes the joint jerk vector, and *T* signifies the total execution duration of the motion. It should be noted that the adopted jerk cost is computed in the joint space by integrating the squared joint jerk throughout the task execution. As an engineering measure of motion smoothness, this metric differs from the classical minimum-jerk hypothesis in human motor control, which is typically formulated in the task space [59]. Therefore, the reported jerk cost might not be suitable for a direct measure of human likeness. The quantitative results are summarized in Table 4, which are obtained from 100 independent trials using each method.

It can be observed that the proposed multi-pathway method achieves a higher success rate and shorter time-to-goal compared to the other methods. This is attributed to the integration and modulation of the fast and slow pathways, allowing the arm to flexibly plan global trajectories and take obstacle-avoidance actions. In contrast, the pure planning methods ${\mathrm{RT-RRT}}_{\mathrm{Smart}}^{*}$ and RT-RRT^*^ require more frequent adjustments when faced with rapidly moving dynamic obstacles, leading to increased instances where the robot hesitates in front of obstacles and fails to reach the goal in time.

The few failures observed in the proposed multi-pathway framework are primarily associated with situations requiring frequent reactive corrections by the fast pathway when dynamic obstacles repeatedly approached the planned trajectory. In these cases, the robot often alternated multiple times between reactive avoidance and goal-directed motion, which could reduce effective task progress and occasionally result in exceeding the 10 s task duration limit. Notably, no failure caused by direct collision with obstacles was observed in the tested scenarios. Qualitative inspection of the failed trials suggests that the absence of explicit obstacle motion prediction in both pathways may limit the coordination between long-horizon planning and short-term reactive avoidance. Incorporating predictive mechanisms to improve pathway cooperation represents a direction for future work.

Furthermore, the performance comparison between RT-RRT^*^ and ${\mathrm{RT-RRT}}_{\mathrm{Smart}}^{*}$ serves as a quantitative ablation analysis establishing the independent impact of the Smart optimization technique on reducing trajectory redundancy. Specifically, with the inclusion of the Smart mechanism, the average planned path length decreases, with an approximate 20% reduction in alignment and improved path quality. Benefiting from fewer redundant paths, ${\mathrm{RT-RRT}}_{\mathrm{Smart}}^{*}$ reduces the situations faced with dynamic obstacles, thus increasing the success rate and achieving superior time to reach the goal compared to RT-RRT^*^.

Meanwhile, the RL-based motion decision-making methods are also tested in repeated experiments, with the same setting as the fast pathway. Although the arms under the RL methods can reach the goal relatively quickly, they exhibit low success rates and longer trajectories with higher redundancy, which may be attributed to the tendency of the robot to attempt aggressive motion to avoid obstacles when approaching them closely, potentially leading to collisions with static obstacles. This underscores the significance of integrating the fast- and slow-pathway methods. In the cases of the RL methods, we additionally calculate the average distance between the end-effector and the goal for 5–10s in tasks (for sampling-based planning methods, since dynamic obstacles do not pass through the target point, the robot stops at the goal after reaching it; hence, this item is not included in the statistics). It can be observed that the presence of obstacles makes it more difficult for the robot to stay at the goal, indicating the necessity of combining RL methods with other approaches in such tasks.

As for motion smoothness, the proposed multi-pathway method achieves a global jerk cost of $5.91\times 10^{3}$, which is substantially lower than those of the reinforcement learning methods. This result indicates that the proposed method generates smoother and more stable motions than the learning-based baselines while maintaining a significantly higher success rate.

Although ${\mathrm{RT-RRT}}_{\mathrm{Smart}}^{*}$ and RT-RRT^*^ exhibit lower global jerk costs, direct comparison requires careful interpretation because the methods adopt different obstacle-avoidance strategies in dynamic environments. Specifically, the planning-based baselines mainly respond to dynamic obstacles through replanning and local waiting, without performing dedicated rapid reactive avoidance maneuvers. Their lower global jerk costs are therefore largely attributable to the absence of such reactive motions rather than superior smoothness under equivalent motion behaviors.

To further distinguish planning smoothness from reactive avoidance behavior, the global jerk cost of the proposed method is further decomposed by integrating the joint jerk separately over the time intervals during which the slow pathway and the fast pathway are active. The results show that the slow-pathway segments yield a jerk cost of $0.73\times 10^{3}$, which is comparable to that of ${\mathrm{RT-RRT}}_{\mathrm{Smart}}^{*}$. In contrast, the fast-pathway activation segments exhibit a jerk cost of $18.2\times 10^{3}$, reflecting the rapid corrective maneuvers required for emergency obstacle avoidance. Consequently, the increased global jerk cost originates primarily from transient reactive avoidance behaviors rather than degraded planning smoothness. These results indicate that the proposed framework effectively balances planning smoothness and reactive responsiveness in dynamic environments.

To assess the statistical significance of the observed performance differences, a chi-square test is conducted on the success rates and a Kruskal–Wallis test is performed on the task completion times. The results indicate that the proposed multi-pathway framework achieves a significantly higher success rate than the original RT-RRT^*^ ($\chi^{2}=4.42$, p<0.05) and SAC ($\chi^{2}=51.19$, p<0.01). As for task time, the test revealed significant differences among the methods (H=175.09, p<0.001). Post hoc pairwise comparisons further confirm that the proposed method achieves significantly shorter times than the other baselines (p<0.001). For the proposed framework, the performance estimates obtained from 100 independent trials also exhibit narrow 95% confidence intervals, with a success rate of 92% (95% CI: 85.0–95.9%), an average task completion time of 4.17 s (95% CI: 4.011–4.329 s), and an average path length of 0.67 m (95% CI: 0.656–0.684 m), indicating satisfactory statistical reliability and repeatability of the reported results.

It should be noted that in the experiments, the reward function design of the RL method considers both obstacle avoidance and goal achievement learning objectives. Adjusting the reward settings may potentially lead to quicker motion towards the goal.

### 5.6. Ablation Study of Modulation Module

To evaluate the contribution of the modulation mechanism to the multi-pathway framework, an ablation experiment is conducted to compare the task performance of the humanoid arm with and without the modulation module. The control group without the modulation module comprises only two pathways, excluding the emotional modulation of the fast pathway on the slow pathway, and employs a bang-bang-like switching based on obstacle distance.

The experimental results suggest that in the absence of the modulation-driven fast-pathway guidance, the overall task success rate shows a slight decrease from 92% to 91%. Meanwhile, the average planning time increases from 4.17±0.80s to 5.42±0.93s, and the average path length grows from 0.67±0.07m to 0.82±0.13m, inferior to that of pure planning-based baseline methods. These results indicate that removing the proposed modulation mechanism leads to a noticeable degradation in planning efficiency and trajectory quality compared to the full model.

**Figure 13:**
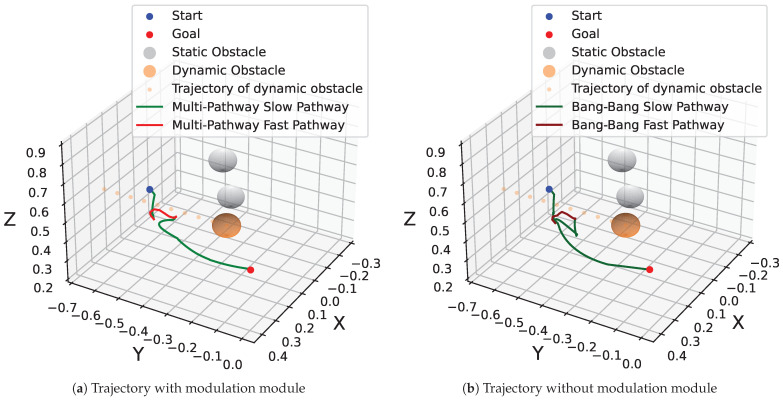
Ablation study evaluating the effectiveness of the proposed modulation mechanism. The comparison highlights the end-effector trajectories of the 6-DoF manipulator under different pathway-switching strategies. (**a**) Trajectory generated using the proposed continuous modulation mechanism, demonstrating smooth and stable transitions between decision-making modes. (**b**) Trajectory generated using a rigid bang-bang switching strategy without continuous modulation, resulting in less efficient trajectory adaptation.

The trajectories of the end-effector under the two circumstances are shown in Figure 13. It can be observed that without the modulation module, the robot tends to revert to its original point where the fast pathway is triggered when transitioning from fast to slow, which is evidently suboptimal and inconsistent with human behavior. In contrast, the modulation module enables the robot to follow a more optimal strategy back to the original planned path. This improvement is attributed to the proposed modulation mechanism, which allows the guidance information from the fast pathway to be incorporated into the sampling process of the slow pathway in a gradual manner. With habitual sampling points, ${\mathrm{RT-RRT}}_{\mathrm{Smart}}^{*}$ can efficiently optimize its planning based on the current state. The above analysis indicates that the proposed modulation mechanism facilitates smoother transitions between the reactive and deliberative decision-making modes, thereby improving planning continuity and trajectory quality.

### 5.7. Parameter Sensitivity Analysis

**Figure 14:**
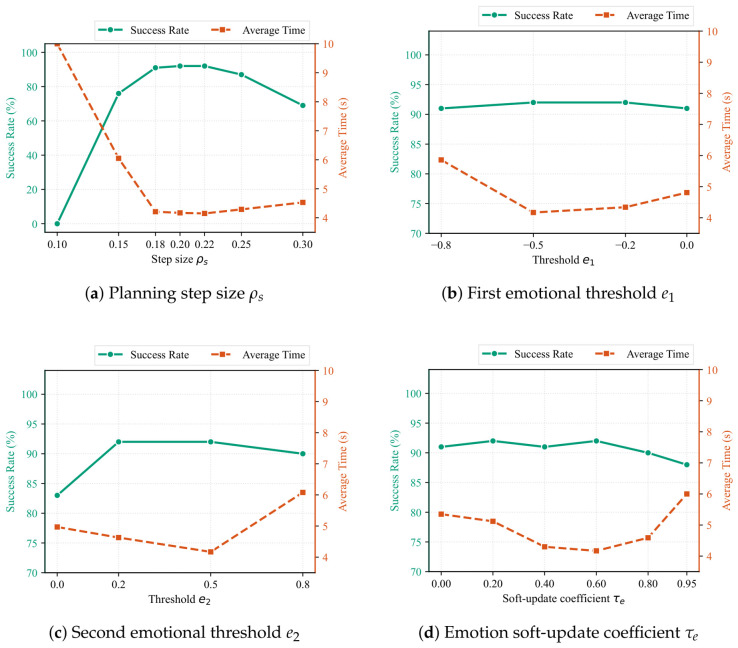
Parameter sensitivity analysis of the proposed multi-pathway framework. The effects of (**a**) the planning step size $\rho_{s}$, (**b**) the first emotional threshold $e_{1}$, (**c**) the second emotional threshold $e_{2}$, and (**d**) the emotion soft-update coefficient $\tau_{e}$ on the task performance are illustrated. The success rate and average task completion time are reported for different parameter values.

To evaluate the sensitivity of the proposed framework to parameter selection, experiments are conducted by varying both the planning parameter and the emotional modulation parameters, while the nominal values correspond to the parameter configuration adopted throughout all the experiments in Table 2. Specifically, the planning step size $\rho_{s}$, the first emotional threshold $e_{1}$, the second emotional threshold $e_{2}$, and the emotion soft-update coefficient $\tau_{e}$ are investigated. The success rate and average task completion time are recorded. The corresponding results are summarized in Figure 14.

As shown in Figure 14a, the planning step size $\rho_{s}$ has a noticeable influence on the overall task performance. Extremely small step sizes lead to excessive node generation and increased computational burden, whereas excessively large step sizes reduce the planning resolution and deteriorate path quality. In particular, when $\rho_{s}=0.10$, the planner generates a substantially larger search tree, causing the planning time to exceed the imposed 10 s limit and resulting in task failure. Conversely, large step sizes reduce the planner’s ability to efficiently explore narrow feasible regions, leading to a gradual decrease in the success rate. Nevertheless, the proposed framework maintains stable performance over a relatively broad range around the nominal value ($\rho_{s}=0.20$), indicating that the planning component is not overly sensitive to moderate variations in this parameter.

Figure 14b–d illustrate the sensitivity of the proposed framework to the emotional modulation parameters. Overall, the framework maintains relatively stable performance over a broad range of parameter values, indicating that satisfactory performance can be achieved without highly precise parameter tuning.

As shown in Figure 14b, the first emotional threshold $e_{1}$ primarily determines the onset of habitual guidance during slow-pathway planning. Variations in $e_{1}$ have only a minor influence on the overall success rate, while planning efficiency is more noticeably affected. When $e_{1}$ is excessively small, the fast pathway is invoked to guide planning even under relatively mild environmental disturbances, resulting in unnecessary guidance and increased task completion time. Conversely, overly large values delay the introduction of habitual guidance, reducing its benefit to the planner and slightly degrading planning efficiency. These observations suggest that the proposed framework is relatively insensitive to moderate variations in $e_{1}$.

The second emotional threshold $e_{2}$, shown in Figure 14c, has a more pronounced influence on the overall task performance because it determines the transition from guidance-assisted planning to fully reactive obstacle avoidance. When $e_{2}$ is set too low, the robot enters the fast pathway prematurely and relies excessively on reactive control, leading to a reduced success rate and increased path length. In contrast, excessively large values delay the activation of the fast pathway, limiting its ability to respond promptly to imminent collision threats and consequently degrading task performance. The value adopted in this work provides a favorable balance between reactive avoidance and deliberative planning.

Figure 14d presents the influence of the emotion soft-update coefficient $\tau_{e}$. Small values allow the emotional state to change rapidly, which may lead to frequent pathway switching and repeated replanning. Conversely, excessively large values slow the adaptation of the emotional state, delaying both the activation and deactivation of the fast pathway. As a result, although the executed trajectories remain relatively compact, the robot requires longer task completion times and exhibits a slight reduction in success rate. The adopted value achieves a suitable compromise between responsiveness and switching stability.

The sensitivity analysis demonstrates that the proposed framework exhibits satisfactory robustness with respect to parameter selection. Among the investigated parameters, the planning step size $\rho_{s}$ and the second emotional threshold $e_{2}$ have comparatively greater influence on task performance, whereas the framework remains relatively insensitive to moderate variations in $e_{1}$ and $\tau_{e}$. These results indicate that the proposed method does not rely on highly precise parameter tuning and can maintain stable performance over a practical range of parameter values.

Overall, the experimental results consistently demonstrate the effectiveness of the proposed multi-pathway framework in dynamic obstacle-avoidance tasks. Compared with the baseline methods, the proposed approach achieves a higher success rate while maintaining competitive planning efficiency and path quality. The results further indicate that the integration of the slow and fast pathways enables the humanoid arm to balance long-horizon planning capability with rapid reactive obstacle avoidance, whereas the proposed modulation mechanism facilitates adaptive pathway arbitration under varying environmental threats. Furthermore, the experimental scenarios are designed to evaluate different aspects of motion decision complexity, including obstacle-constrained reaching in mixed static–dynamic environments and rapid evasive maneuvers under dynamic projectile threats. These complementary experiments suggest that the proposed framework is capable of operating under both structured and dynamic conditions. Nevertheless, the current study considers a limited set of obstacle configurations and motion patterns. Evaluations in more densely cluttered environments and under more diverse dynamic behaviors will be investigated in future work.

## 6. Conclusions

This paper presents a biologically inspired multi-pathway framework for humanoid arm motion decision-making in dynamic environments. The proposed framework adopts functional abstractions inspired by the PFC, striatum, and amygdala to enable multi-pathway motion decision-making for humanoid arms, coordinating deliberative planning, rapid reactive avoidance, and adaptive pathway switching. The experimental results demonstrate that the proposed framework effectively combines the long-horizon planning capability of sampling-based methods with the rapid responsiveness of reinforcement learning policies. Comparative studies show that this improved sampling strategy reduces path redundancy, while the integration of the two pathways improves task success rate and shortens overall task completion time. Furthermore, the proposed modulation mechanism enables adaptive coordination between the planning and reactive pathways, contributing to the robustness and adaptability of the overall decision-making framework.

The potential applications of this research extend across industrial robot operations, human–robot collaborative tasks, and co-working scenarios, basically enhancing the safety of robotic operations. Hopefully, the multi-pathway motion decision-making method is to be further applied to dual-arm motion, actual operations, and HRI tasks. In future work, additional biologically inspired computational principles will be investigated to further enhance the predictive planning capability of the proposed framework. Moreover, the reactive adaptation capability of the fast pathway could be further improved by incorporating hybrid reinforcement learning frameworks, such as integrating fuzzy logic with reinforcement learning agents [60], enabling more adaptive modulation of safety boundaries according to the instantaneous motion of dynamic obstacles. Furthermore, practical deployment may be affected by sim-to-real discrepancies arising from actuator dynamics, transmission effects, sensor noise, state-estimation uncertainty, and unmodeled structural flexibility. Future work will therefore focus on hardware validation, sim-to-real adaptation, and the extension of the proposed framework to higher-dimensional robotic platforms, including dual-arm manipulation and human–robot interaction scenarios.

## Figures and Tables

**Table 1:** Parameters for fast-pathway SAC training.

Name	Notation	Value
Hidden layer size of actor network	$N_{h}^{\pi }$	256
Hidden layer size of critic network	$N_{h}^{Q}$	256
Actor learning rate	$\eta_{\pi }$	0.0003
Critic learning rate	$\eta_{Q}$	0.0001
Replay pool size	$\left|𝒟\right|$	100,000
Training episodes	$N_{\mathrm{episode}}$	310
Maximum time steps per episode	$N_{\mathrm{step}}$	1000
Soft update factor	τ	0.01
Sampling batch size	$N_{\mathrm{batch}}$	128
Discount factor	γ	0.9
Survival reward factor	$r_{s}$	500
Weight of reward factors	$w_{r}$	[100, 20, 100, 20]^T^
Collision penalty	$r_{c}$	5
Joint velocity bound	${\dot{q}}_{\mathrm{bound}}$	[−1, 1]^T^
Joint acceleration bound	${\ddot{q}}_{\mathrm{bound}}$	[−10, 10]^T^
Referenced distance for $R_{o}$	$d_{\mathrm{ref}}$	0.1

**Table 2:** Parameters for slow pathway and modulation module.

Name	Notation	Value
Step size	$\rho_{s}$	0.2
Neighbor radius	$\rho_{N}$	0.3
Probability to sample goal	$p_{g}$	0.3
Probability to sample in ellipse	$p_{e}$	0.3
Path planning weight	λ	1
Period for control	$T_{c}$	0.05
Period for planning	$T_{d}$	0.2
Trajectory planning density	$k_{ra}$	200
Size of LA layer	$N_{\mathrm{LA}}$	64
Size of BA layer	$N_{\mathrm{BA}}$	32
Size of ITC layer	$N_{\mathrm{ITC}}$	32
Size of CeM layer	$N_{\mathrm{CeM}}$	1
Emotional threshold 1	$e_{1}$	−0.5
Emotional threshold 2	$e_{2}$	0.5
Weight in EmoNet Training Loss	$w_{e}$	[1, 1]^T^
Reference distance of $e_{d}$	$d_{f}$	0.1
Maximum probability of $p_{f}$	$p_{fm}$	0.8
Reference radius of $f_{\mathrm{fast}}$ sampling	$\rho_{f0}$	0.6
Coefficient of emotion soft-update	$\tau_{e}$	0.6

**Table 3:** Runtime profiling of the slow pathway under different nearest-neighbor search methods and tree sizes (unit: ms).

Computation Module	HNSW			KD-Tree			Brute Force		
	1000	2000	4000	1000	2000	4000	1000	2000	4000
EmoNet inference	<1.0	<1.0	<1.0	<1.0	<1.0	<1.0	<1.0	<1.0	<1.0
SAC inference	<1.0	<1.0	<1.0	<1.0	<1.0	<1.0	<1.0	<1.0	<1.0
Nearest-neighbor query	9.0	8.9	18.3	5.8	6.4	13.7	257.7	650.1	1790.6
Collision checking	67.9	77.3	124.9	83.6	121.6	293.3	70.9	135.0	217.2
TOPP-RA	12.8	14.5	18.2	13.0	14.4	18.1	12.8	14.6	17.9
Overall planning cycle	91.7	102.8	163.3	104.4	144.4	327.1	343.4	801.7	2027.7

**Table 4:** Performance comparison of algorithms.

Method	Success Rate	Average Time (s)	Average Path Length (m)	Jerk Cost ($\times 10^{3}$)	Average Distance to Goal in 5–10 s (m)
Multi-Pathway	92%	4.17±0.80	0.67±0.07	5.91±0.65	−
${\mathrm{RT-RRT}}_{\mathrm{Smart}}^{*}$	88%	6.11±0.94	0.58±0.09	0.72±0.06	−
RT-RRT^*^	82%	7.04±1.54	0.73±0.12	0.81±0.05	−
SAC	45%	5.03±2.50	1.24±0.45	11.0±1.2	0.24±0.14
TD3	25%	4.75±2.26	1.98±0.39	17.3±2.1	0.25±0.15
DDPG	18%	5.31±2.71	2.04±0.19	9.8±1.3	0.31±0.13

**Figure A1:**
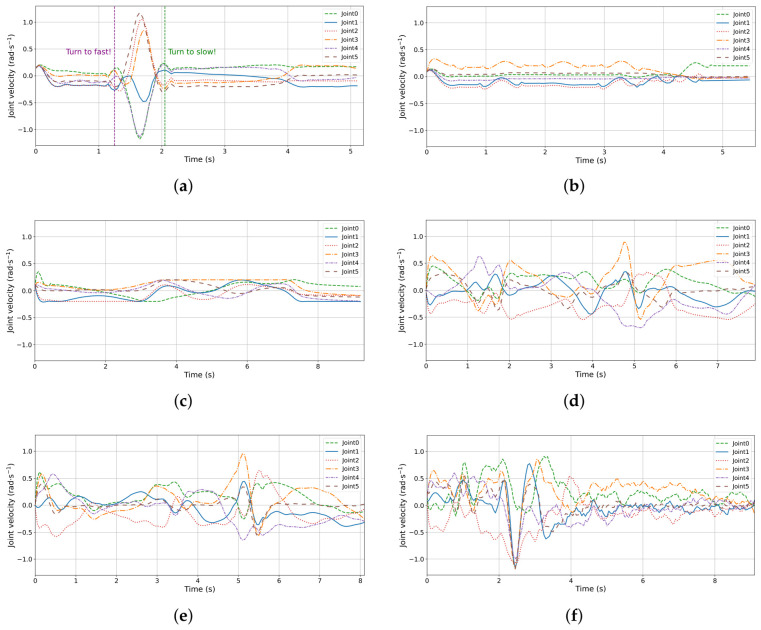
Joint velocity trajectories of the 6-DOF humanoid arm under different methods in the goal-reaching task with obstacles. (**a**) Multi-Pathway. (**b**) ${\mathrm{RT-RRT}}_{\mathrm{Smart}}^{*}$. (**c**) RT-RRT^*^. (**d**) SAC. (**e**) DDPG. (**f**) TD3.

## Data Availability

The original contributions presented in this study are included in the article. Further inquiries can be directed to the corresponding author.

## Abbreviations

The following abbreviations are used in this manuscript:

HRI	Human–Robot Interaction
RL	Reinforcement Learning
MFRL	Model-Free Reinforcement Learning
PFC	Prefrontal Cortex
dlPFC	Dorsolateral Prefrontal Cortex
vmPFC	Ventromedial Prefrontal Cortex
OFC	Orbitofrontal Cortex
DOF	Degree of Freedom
RRT	Rapidly exploring Random Tree
RRT^∗^	Rapidly exploring Random Tree Star
RT-RRT^*^	Real-Time Rapidly exploring Random Tree Star
BFS	Breadth-First Search
NN	Neural Network
MDP	Markov Decision Process
SAC	Soft Actor-Critic
ReLU	Rectified Linear Unit
TTR	Time-to-Reach
NNS	Nearest-Neighbor Search
HNSW	Hierarchical Navigable Small World
TOPP-RA	Time-Optimal Path Parameterization based on Reachability Analysis
FSM	Finite State Machine
MLP	Multilayer Perceptron
LA	Lateral Amygdala
BA	Basal Amygdala
CeM	Central Amygdala
ITCs	Intercalated Cell Clusters
CS	Conditioned Stimulus
US	Unconditioned Stimulus
URDF	Unified Robot Description Format
MSE	Mean Squared Error
DDPG	Deep Deterministic Policy Gradient
TD3	Twin Delayed Deep Deterministic Policy Gradient

## Appendix A. Notation and Symbol Definitions

This appendix summarizes the key mathematical symbols used throughout the paper, organized according to their first appearance in different sections.

**Table A1:** Symbols Introduced in Section II-A: Problem Definition.

Symbol	Description
𝒳	Planning state space, $𝒳\subseteq R^{m}$, m∈N.
${𝒳}_{\mathrm{obs}}$	Set of all obstacles within the space.
${𝒳}_{\mathrm{free}}$	Free space, defined as ${𝒳}_{\mathrm{free}}=𝒳\setminus {𝒳}_{\mathrm{obs}}$.
x	A state of the agent, x∈𝒳.
$x_{\mathrm{init}}$	Initial state of the agent.
$x_{\mathrm{goal}}$	Goal state of the agent.
$x_{a}$	Current state of the agent.
$t_{0}$	Planning start time.
σ(s)	A trajectory. s∈[0,1]. σ:[0,1]→𝒳
Σ	Set of all possible trajectories. σ∈Σ.
c(σ)	Cost function evaluating trajectory quality. c:Σ→R.
$\sigma^{*}$	Optimal trajectory.

**Table A2:** Symbols Introduced in Section II-B: Sampling-based Method.

Symbol	Description
$\sigma_{p}$	Finite path sequence $\langle x_{0},x_{1},\ldots ,x_{k}\rangle$.
$d(x_{i},x_{j})$	Distance metric between $x_{i}$ and $x_{j}$.
$c(\sigma_{p})$	Cost function of path $\sigma_{p}$.
$c(x_{i})$	Cost of path $\sigma_{p}\left(x_{i}\right)$.
ㄒ	Search tree used in RRT algorithms, defined as ㄒ=(𝒱,E).
𝒱	Set of nodes (vertices) in the search tree, 𝒱⊂𝒳.
E	Set of weighted edges in the tree, E:𝒱×𝒱→R.
$x_{\mathrm{root}}$	Root node of the search tree ㄒ.
$\sigma_{p}\left(x_{i}\right)$	Unique path from root $x_{\mathrm{root}}$ to node $x_{i}$ in the tree.
$c(x_{i})$	Cost of the path $\sigma_{p}\left(x_{i}\right)$ from $x_{\mathrm{root}}$ to $x_{i}$.
$x_{\mathrm{rand}}$	Randomly sampled state from a sampling distribution function.
f(x,ㄒ)	Sampling distribution function depending on current state and the search tree.
$x_{\mathrm{nearest}}$	The nearest node in the tree to the sampled state $x_{\mathrm{rand}}$.
$x_{\mathrm{new}}$	New node generated from $x_{\mathrm{nearest}}$ toward $x_{\mathrm{rand}}$.
$\rho_{s}$	Maximum step size for steering toward the sampled state.
$\rho_{N}$	Neighborhood radius for finding nearby nodes around a new node.
$x_{\min}$	Minimum-cost parent node selected from the neighborhood of $x_{\mathrm{new}}$.
${𝒳}_{\mathrm{near}}$	Set of nodes within distance $\rho_{N}$ from a given node.
$Q_{r}$	Queue used in Rewire_new_ to manage update order of affected nodes.
$Q_{s}$	Queue used in Rewire_root_ to manage update order near the tree root.
$T_{d}$	Duration of each online planning update cycle.
$t_{\mathrm{plan}}$	Remaining planning time allowed in the current update cycle.

**Table A3:** Symbols Introduced in Section III-A: RL-based Method.

Symbol	Description
*M*	MDP tuple used to model the decision-making process. M=(𝒮,𝒜,P,r,γ).
𝒮	State space of MDP, defined as 𝒮=[𝒳,Z].
Z	Additional environment-related state components beyond 𝒳.
𝒜	Action space.
*P*	State transition function. P:𝒮×𝒜→𝒮.
*r*	Reward function. r:𝒮×𝒜→R.
γ	Discount factor in RL, γ∈[0,1).
$s_{t}$	State at time step *t*. $s_{t}\in 𝒮$.
$a_{t}$	Action taken at time step *t*. $a_{t}\in 𝒜$.
π	Policy mapping states to action distributions.
$\pi^{*}$	Optimal policy.
$\sigma_{n}$	Discretized trajectory sequence indexed by *n*.
$T_{c}$	Control period for discrete trajectory generation.
$t_{f}$	Execution finishing time of the trajectory.
H(π)	Entropy of policy π, used to quantifies the uncertainty of the policy.
α	Temperature coefficient balancing reward and entropy in SAC.
θ	Parameters of the critic network.
ϕ	Parameters of the actor network.
$Q_{\theta }$	Action-value function (critic) parameterized by θ.
$\pi_{\phi }$	Policy (actor) parameterized by ϕ.
$J_{\pi }\left(\phi \right)$	Objective function used to update the actor network parameters.
$J_{Q}\left(\theta \right)$	Objective function used to update the critic network parameters.
$V(s_{t})$	Soft value function defined using *Q* and entropy regularization.
$V_{\theta }\left(s_{t}\right)$	Soft value function implicitly parameterized via $Q_{\theta }$.
𝒟	Sample space or replay buffer used for training.
J(α)	Loss function for adapting α.
$H_{0}$	Target entropy, defined as $H_{0}=-\dim\left(𝒜\right)$.

**Table A4:** Symbols Introduced in Section III-B: The Fast-Pathway Motion.

Symbol	Description
q	Joint position of the robot.
$p_{\mathrm{goal}}$	Desired goal position in task space.
$d_{o}$	Minimum distance between the robot and obstacles in task space.
$p_{rd}$	The point on the robot closest to the obstacle.
$p_{od}$	The point on the obstacle closest to the robot.
$\theta_{i}$	Parameters of the *i*-th critic network (i=1,2).
${\bar{\theta }}_{i}$	Parameters of the *i*-th target critic network.
τ	Soft update rate for target network parameter updates.
π	Policy function represented by the actor network.
$w_{r}$	Weight vector for different reward components.
$r_{s}$	Scalar survival reward encouraging longer survival.
$r_{g}$	Reward term based on distance between the end-effector and target.
$p_{ee}$	Position of the robot end-effector in task space.
$r_{a}$	Penalty term based on the amplitude of the action.
$r_{o}$	Reward related to obstacle distance, penalizing potential collisions.
$r_{c}$	Penalty constant applied when a collision occurs.
$d_{\mathrm{ref}}$	Reference distance parameter controlling $r_{o}$ shape.
$r_{p}$	Penalty for action exceeding predefined range constraints.

**Table A5:** Symbols Introduced in Section III-C: Slow-pathway Planning.

Symbol	Description
𝒞	Joint space of the robot with *m* degrees of freedom. $𝒞\subseteq R^{m}$.
𝒲	Task space (workspace) in which the robot and obstacles are represented.
M(x)	Robot’s shape or occupancy in task space 𝒲 under configuration x.
𝒪	Representation of obstacles task space 𝒲.
$f_{\mathrm{fast}}$	Sampling distribution guided by the fast pathway actor policy π.
$f_{r}$	Residual sampling strategy independent of the fast pathway.
$p_{f}$, $p_{g}$, $p_{e}$	Probabilities determining the selection of different sampling strategies.
I(·)	Indicator function, selects a specific point deterministically.
U(·)	Uniform distribution function over a given set.
B	Hyperellipsoid region, used for informed sampling.
$a_{e}$	Transverse diameter of the hyperellipsoid B.
$b_{e}$	Conjugate diameter of the hyperellipsoid B.
*k*	The number of nearest neighbors returned in HNSW query for Nearest and Near.
$k_{N}$	Maximum number of neighbor nodes considered within radius $\rho_{N}$ during Near query.
$x_{p}$	A temporary node selected during planning when $x_{\mathrm{goal}}$ is not reached, minimizing the weighted cost and distance to goal.
λ	Weight factor that balances cost-to-come and distance-to-goal in selecting $x_{p}$.
$x_{S}$	The newly computed Smart path node generated by $\text{Steer}(x_{\mathrm{root}},x_{1})$ to maintain path feasibility.
$𝒢(x_{\mathrm{goal}})$	The goal region in state space 𝒳 indicating that ㄒ has reached $x_{\mathrm{goal}}$.
$\sigma_{pa}$	Augmented path composed of initial point and planned path: $\sigma_{pa}=\left\{x_{a}\right\}\cup \sigma_{p}\left(ㄒ\right)$.
$N_{pa}$	Number of points in the augmented path
$x_{pa}^{i}$	The *i*-th point on the augmented path, $i=0,1,\ldots ,N_{pa}$.
$\sigma_{i}\left(s\right)$	Cubic spline trajectory on the interval $\left[s_{i-1},s_{i}\right]$, parameterized by *s*.
$h_{il}$	Coefficient vector of the cubic spline segment $\sigma_{i}$, where l=0,1,2,3.
$s_{i}$	Monotonically increasing knot sequence for the spline, defined on [0,1].
$s_{pa}^{i}$	Normalized arc length parameter for trajectory discretization.
$u_{i}$	Action (control input) at index *i* along the trajectory.
$N_{ra}$	Number of discretized trajectory steps used in TOPP-RA, proportional to the trajectory length.
$k_{ra}$	Proportional constant for determining $N_{ra}$.
$K_{i}$	Controllable set at step *i* along the trajectory.
$\Theta_{i}\left(K\right)$	One-step set reachable from set K at step *i*.
$\Delta_{i}$	Discrete interval between spline parameters. $\Delta_{i}=s_{i+1}-s_{i}$.
$\Omega_{i}$	Feasible set for the action–state pair $\left(u_{i},x_{i}\right)$ under constraints.
$x_{i}^{*}$	Optimized state at step *i* from forward pass in TOPP-RA.
$u_{i}^{*}$	Optimal action at step *i* from forward pass in TOPP-RA.

**Table A6:** Symbols Introduced in Section III-D: Emotion-based Multi-Pathway Modulation.

Symbol	Description
$e_{1}$, $e_{2}$	Emotional thresholds for switching between slow and fast pathways.
$p_{\mathrm{dyo}}$, ${\dot{p}}_{\mathrm{dyo}}$	Position and velocity of dynamic obstacle in task space.
$I_{\mathrm{collision}}$	Indicator function of a collision event.
*e*	Output emotional signal from EmoNet.
$\theta_{e}$	Parameter (weights) of EmoNet neural network.
$e_{d}$	Emotion-related measure based on distance to dynamic obstacle.
$d_{\mathrm{dyo}}$	Distance to the dynamic obstacle.
$w_{e}$	Weight vector balancing $e_{d}$ and $I_{\mathrm{collision}}$ in emotion output.
$\zeta_{d}$	Coefficient for exponential decay in $e_{d}$ computation.
$d_{f}$	Reference distance to obstacle associated with $e_{2}$.
$p_{f}$	Probability of sampling guided by fast pathway.
$p_{fm}$	Maximum value of $p_{f}$ when $e=e_{2}$.
$f_{\mathrm{fast}}\left(\cdot \right)$	Sampling function under fast-pathway guidance.
$x_{f}$	Sampled planning state along the trajectory σ near $x_{a}$.
$\rho_{f}$	Radius for sampling near $x_{a}$ under emotion modulation.
$\rho_{f0}$	Base sampling radius in $\rho_{f}$ definition.
$s_{f}$	State description at $x_{f}$ including velocity and environment info.
$\rho_{S}$	Step size in sampling toward $\pi (s_{f})$ direction.
$\tau_{e}$	Coefficient for soft-update of emotion signal.

## Appendix B. Detailed Results and Comparison of Joint Trajectories Under Different Methods in Goal-Reaching Task

This appendix provides additional results to supplement the main text by presenting and comparing the velocity trajectories of all robot joints with different methods in the goal-reaching experiments, thereby offering a more comprehensive demonstration of the effectiveness, rationality, progressiveness, and accuracy of the proposed approach.

As described in the main text, a 6-DoF humanoid manipulator is employed to simulate human arm motion. The robot is required to accomplish a goal-reaching task in an environment containing both dynamic and static obstacles. Specifically, the experimental setup consists of two stationary obstacles and one periodically moving obstacle, which oscillates between points [0.3, −0.15, 0.5]^T^ and [0.3, −0.65, 0.5]^T^ near the target. The arm starts from the initial posture $q_{\mathrm{init}}=\;$[0.838, 1.082, 1.553, −1.187, 1.571, 0]^T^ and move toward the target end-effector position $p_{\mathrm{goal}}=\;$[0.4, 0, 0.5]^T^.

Figure A1 presents the representative joint velocity trajectories obtained under six different methods in the goal-reaching task with mixed static and dynamic obstacles. In all depicted experiments, the robot successfully avoided collisions and reached the designated target.

For planning-based methods, the arm is generally able to avoid static obstacles and smoothly moving dynamic obstacles in a stable and safe manner, and successfully reach the target. After arrival, since the dynamic obstacle does not threaten the goal region and the target remains feasible in the planning formulation, the arm is able to stably remain at the target position. Notably, in the proposed method Multi-Pathway, the joint velocity exhibits a distinct adjustment when the dynamic obstacle approaches, characterized by a rapid increase followed by a decrease. This reflects the ability of the humanoid arm to generate a prompt avoidance action while executing the planning motion. By contrast, under ${\mathrm{RT-RRT}}_{\mathrm{Smart}}^{*}$, the absence of a dedicated fast-path avoidance mechanism leads the robot to perform multiple avoidance maneuvers (e.g., at approximately 1.3 s and 3.3 s), which are smoother but slower, thereby reducing its effectiveness in handling sudden obstacle intrusions. In the case of RT-RRT^*^, where the SMART heuristic is not applied, the velocity variations are more frequent, resulting in redundant and less efficient trajectories compared with ${\mathrm{RT-RRT}}_{\mathrm{Smart}}^{*}$.

For RL–based methods, the velocity trajectories are more sensitive to changes according to the distance from obstacles, as the learned policies are optimized with respect to value functions that explicitly encode obstacle proximity. This sensitivity causes larger fluctuations in the joint motions compared with planning-based methods. In particular, both SAC and TD3 exhibit multiple oscillations in joint velocities, leading to repeated avoidance maneuvers, which highlights their suitability for learning obstacle-avoidance strategies but also indicates a lack of stability in convergence toward the goal. Under DDPG, the robot executes a pronounced avoidance maneuver at around 2.4 s, yet maintaining a relatively stable velocity profile for the remainder of the task, albeit with noticeable fluctuations.

Overall, these comparisons demonstrate that the proposed multi-pathway framework achieves a balance between the stability of planning-based approaches and the responsiveness of reinforcement learning–based strategies, thereby enabling efficient and human-like obstacle avoidance while ensuring successful task completion.
